# Scientific Wealth in Middle East and North Africa: Productivity, Indigeneity, and Specialty in 1981–2013

**DOI:** 10.1371/journal.pone.0164500

**Published:** 2016-11-07

**Authors:** Afreen Siddiqi, Jonathan Stoppani, Laura Diaz Anadon, Venkatesh Narayanamurti

**Affiliations:** 1 Belfer Center for Science and International Affairs, John F. Kennedy School of Government, Harvard University, Cambridge, Massachusetts, United States of America; 2 Institute for Data, Systems, and Society, Massachusetts Institute of Technology, Cambridge, Massachusetts, United States of America; 3 Haute école spécialisée de Suisse occidentale, Boulevard de Perolles 80, 1700, Fribourg, Switzerland; 4 Department of Politics and International Studies, University of Cambridge, Cambridge, United Kingdom; 5 School of Engineering and Applied Sciences, Harvard University, Cambridge, Massachusetts, United States of America; Iowa State University, UNITED STATES

## Abstract

Several developing countries seek to build knowledge-based economies by attempting to expand scientific research capabilities. Characterizing the state and direction of progress in this arena is challenging but important. Here, we employ three metrics: a classical metric of productivity (publications per person), an adapted metric which we denote as Revealed Scientific Advantage (developed from work used to compare publications in scientific fields among countries) to characterize disciplinary specialty, and a new metric, scientific indigeneity (defined as the ratio of publications with domestic corresponding authors) to characterize the locus of scientific activity that also serves as a partial proxy for local absorptive capacity. These metrics—using population and publications data that are available for most countries–allow the characterization of some key features of national scientific enterprise. The trends in productivity and indigeneity when compared across other countries and regions can serve as indicators of strength or fragility in the national research ecosystems, and the trends in specialty can allow regional policy makers to assess the extent to which the areas of focus of research align (or not align) with regional priorities. We apply the metrics to study the Middle East and North Africa (MENA)—a region where science and technology capacity will play a key role in national economic diversification. We analyze 9.8 million publication records between 1981–2013 in 17 countries of MENA from Morocco to Iraq and compare it to selected countries throughout the world. The results show that international collaborators increasingly drove the scientific activity in MENA. The median indigeneity reached 52% in 2013 (indicating that almost half of the corresponding authors were located in foreign countries). Additionally, the regional disciplinary focus in chemical and petroleum engineering is waning with modest growth in the life sciences. We find repeated patterns of stagnation and contraction of scientific activity for several MENA countries contributing to a widening productivity gap on an international comparative yardstick. The results prompt questions about the strength of the developing scientific enterprise and highlight the need for consistent long-term policy for effectively addressing regional challenges with domestic research.

## Introduction

The scientific wealth of nations is of critical importance [1], and prevailing theories of growth identify technological innovation as a key driver for economic dynamism and competitive strength [2–4]. Several developing countries are seeking to advance national capabilities in research and innovation, with the ultimate aim of fostering economic diversification and growth. Many experts studying the role of scientific research and technological innovation in broader national development note that countries need an initial (threshold) level of scientific and technological capabilities (*i*.*e*. absorptive capacity) in order to reap the full value of external information, and technology flows [5–6]. Some developing countries seek to advance local infrastructure, education, health, and other important sectors, by importing modern technologies; but for successful adoption, implementation, and use of such technologies, these countries need to have a certain level of internal (domestic) technical ability and capacity for creating new knowledge [7].

Policy makers in several countries, including countries in South East Asia, Middle East, and Africa, have been seeking a transition to the so-called *knowledge-based economies* (OECD, 1996) for the past few decades. For countries that have made efforts to promote domestic research and innovation capacity and economic diversification—through investments in science and technology capacity—a study of how their national scientific enterprise has evolved can help design future policy initiatives. The results can further inform global understanding of and challenges in scientific development in the 21^st^ century–an era of increased globalization marked with an ever-quickening pace of scientific activities and output.

One difficulty in characterizing the state and direction of progress in the scientific arena for countries that had historically low levels of scientific research is lack of detailed data. Some countries, including OECD nations and a subset of other countries, have put in place systems for collecting, compiling, and publishing detailed data about their research enterprise, including national assessment reports regarding the state of research. Many countries in Africa, the Middle East, and elsewhere, however, do not yet have readily accessible, verifiable, and comparable data sources. In some cases, data on investments is being made available (such as through the World Bank’s Data Bank), however complete historical time-series data is rare. These limitations make it difficult to use metrics of input and efficiency (that use ratios of R&D investments and patents and citations) to gauge national performance in science and technology [8] over time.

Here, we use three quantitative metrics: (a) a classical metric of productivity (publications per person) first used by Lotka in 1926, (b) an adapted metric which we denote as Revealed Scientific Advantage, to compare publications in scientific disciplines among countries, and (c) a new metric, scientific indigeneity (defined as the ratio of publications with domestic corresponding authors), to characterize the locus of scientific activity that may also serve as a partial indicator for local absorptive capacity.

These metrics, which rely on population and publications data that are available for most countries over time, allow the characterization of some key features of trends in national scientific enterprise. The temporal productivity and indigeneity when compared across other countries and regions can serve as indicators of strength or fragility in the national research ecosystems, and the trends in specialty can allow regional policy makers to assess the extent to which the areas of focus of research align (or not align) with regional priorities.

We apply the metrics to study the Middle East and North Africa (MENA). Several countries in the region–facing mounting socio-economic challenges with rapidly increasing populations and declining oil reserves—are seeking to advance their research and innovation capabilities [9,10]. Egypt launched the Decade for Science and Technology 2007–16 to strengthen national science and technology. The oil-dependent economies of Saudi Arabia, Qatar, and United Arab Emirates (UAE) have poured billions of dollars since the late 1990s in establishing new universities, creating technology parks, and actively recruiting top researchers around the globe to relocate to the region, as part of efforts for future economic diversification and expansion (Sections A and B in S1 File).

Current data shows that the number of annual scientific publications in the region has risen (Figure A in Section B in S1 File). However, there is a lack of knowledge about how the regional trends compare in an international context and what if any insights maybe useful for the global scientific community. Here we analyze three interconnected questions utilizing newly available data and new indicators: 1) How has national research productivity evolved in MENA and how does it compare with other countries? 2) What has been the contribution of international collaborations on stimulating regional scientific output? 3) What are the areas of disciplinary focus and how do they compare globally?

We investigate these questions of productivity, geography, and disciplinary focus in scientific activities in 17 countries of the MENA region (Morocco, Libya, Algeria, Tunisia, Sudan, Egypt, Jordan, Lebanon, Syria, Saudi Arabia, Yemen, United Arab Emirates, Oman, Bahrain, Qatar, Kuwait, and Iraq) between 1981 and 2013 using publications as a partial indicator of scientific output and conducting an empirical analysis of over 9.84 million records sourced from the Science Citation Index-Expanded. The quantitative long time-series analysis–which has not been previously conducted for the region or for comparison purposes—allowed for characterizing the emergent trends. Additionally, we compared the MENA region with a group of seven carefully selected countries: Turkey (a newly industrializing and emerging economy, geographically and historically connected with MENA), Norway (an industrialized, European welfare economy with extensive North Sea oil reserves), South Korea (an industrialized East Asian economy with consistently one of the highest economic growth rates), Singapore (a trade-oriented economy with strong focus on education and research), Australia (one of the top 20 economies with extensive mining and agricultural sectors), and China (a new global power in manufacturing with increasing scientific research). We also included the US to provide comparison with the globally dominant scientific enterprise.

While the outputs of scientific activities go beyond published papers, bibliometric analysis nevertheless allows for comparisons and quantitative measurements of the system that are traceable over time. The issue of quality of the research output is critical and is an important part of assessing national scientific impact [11]. However, here we focus on quantity noting that for countries that are trying to ramp up their research system, the quantity of peer-reviewed scientific publications in English provides a useful metric of progress given the very recent and major efforts to expand in this area. Citations have their own limitations [12]. Furthermore, the Web of Science citation data does not capture all the relevant citations for decision makers in the region, which include policy documents and reports.

Our quantitative analysis was directed and the results were interpreted in light of over 80 semi-structured interviews that we conducted with faculty, senior administrators (including university presidents and college deans) and technology firm executives during field visits to Morocco, Jordan, Lebanon, Turkey, Kuwait, Saudi Arabia, Qatar, and UAE from 2013–2015 (Section A in S1 File). Our on-site discussions with researchers in the region serve as an important distinguishing factor of this work and informed both our selection and development of quantitative indicators and the interpretation of the quantitative results.

In the following sections we provide a description of the data collection method, a definition and discussion of the metrics we used in this work, and a discussion of key trends at national and regional level that emerge from our analysis along with implications for policy and further research.

## Materials and Methods

We obtained publications data from the Science Citation Index-Expanded through the Web of Science^TM^ Core Collection (Section A in S1 File). The search queries were performed during 2014 through 2015, therefore due to the consistent growth of journals indexed in the database, it is likely that there may be some differences in exact publication counts for queries executed at a later time. The focus of our work was on analyzing total scientific publications in nations that have not had significant research activities in the recent past. Therefore, we chose the Science Citation Index-Expanded (rather than the more widely used Science Citation Index) since it has wider coverage (although of varying journal quality). We included full journal articles (see Table C in S1 File) only and did not account for letters, reviews, conference papers, books or other publications. The yearly population data was obtained from the Data Bank web portal of the World Bank. It was used for computing per capita annual publications for each country (the population data used for 2013 is shown in Table B in S1 File).

### Author Location Data

For author location data, we obtained the full citation records of all publications between 1981–2013 for Saudi Arabia, UAE, Bahrain, Qatar, Kuwait, Oman, Yemen, Iraq, Syria, Sudan and Libya. For rest of the countries (with larger number of publications), we estimated indigeneity using statistical sampling of records for each year and analyzing address information of reprint authors using our text parsing routines coded in Matlab^TM^. The robustness of the results was evaluated and is provided in detail for each country (Table D in S1 File).

### Subject Area Data

We obtained subject area data for each country by analyzing the country results in multi- year intervals (1981–85, 1986–90, 1991–95, 1996–2000, 2001–2005, 2006–2010, and 2011–2013) with research area analysis in Web of Science. This provides count of papers for each area. It should be noted that a paper maybe assigned multiple subject areas (*e*.*g*. it may have two areas such as Ecology and Marine & Freshwater Biology) associated with it. We conducted this search for 175 areas that we determined to be relevant leaving out areas from social sciences (such as economics and business management). The subject areas were consolidated into 15 categories (Table A in S1 File) based on results reported in [13], wherein a systematic decomposition of a journal-journal citation matrix was used to identify inter-connected disciplines. We used those results with some modifications for engineering disciplines relevant for the regional economies.

### Publications Volume

The total publications for a country *i* in year *t* was defined as:

$$X_{i}\left(t\right)=\text{\#of publications with at least one author address in country i}$$ (1)

The whole-counting approach was used. If a publication had three co-authors, and one of the co-authors had an address in Kuwait, the publication would be included as a full count for Kuwait. This approach provided an upper limit accounting of the publications for each country. The attribution for each country was made only on the basis of address information, and the citizenship or national origin of authors was not taken into account. The global share of each country was computed for each year *t* as:

$$\text{Share of country i in world publications}\;\left(\text{t}\right)=\frac{X_{i}\left(t\right)}{\sum_{i=1}^{N}X_{i}\left(t\right)}$$ (2)

*N* is the number of countries with journal publications records in year *t*.

### Scientific Productivity

From Lotka’s pioneering work from almost a century ago, in which he analyzed patterns of scientific productivity for chemists and physicists [14], to current work in scientometrics [15], patterns in research productivity have been continually examined. Productivity (defined as per capita scientific publications) is an important measure when considering training and producing researchers [1]. Here, we measured productivity as the ratio of annual publications and population for each country and computed the scientific productivity, *η*_*i*_ of country *i* in year *t*, as:

$$\eta_{i}\left(t\right)=\frac{X_{i}\left(t\right)}{P_{i}\left(t\right)}$$ (3)

*P*_*i*_*(t)* is population of country *i* in year *t*.

This ratio (of total publications to total population) has been used historically [1–2]. Ideally, the number of total scientists and researchers should be used instead of total population of a country. The annual data of total researchers in each MENA country over the last thirty years, however, is not available. Given this limitation, the total population serves as a proxy variable for determining productivity. This allowed for a common basis of comparison with other countries outside the region, however, it also includes the structural differences in the fraction of population devoted to research.

### Scientific Indigeneity

The issue of location is paramount when accounting for national scientific research activity–particularly when the aim is to investigate the development of local scientific capacity. Prior research has shown that knowledge flows (measured by patent citations) tend to stay geographically localized due to the positive influence of spatial proximity on knowledge sharing and interpersonal relationships [16]. In the age of globalization, national systems of innovation can become more relevant, not less [17], as domestic institutions (shaped by policy) improve competitiveness through building absorptive and innovative capacity and attracting and leveraging resources that are increasingly global. It has been shown that while simple knowledge diffuses equally to close and distant actors, socially proximate recipients have the greatest advantage over distant actors for knowledge of moderate complexity [18], and co-location is also important for firm to university collaborations [19].

Trends in scientific research teams [20], and international collaboration patterns have been investigated extensively, with results showing a growing fraction of scientific research being undertaken by teams of collaborators across different universities [21] and from multiple countries [22]. This research did not investigate differences that may arise by country.

In recognition of the globalization trends, and with the aim of getting insights relevant for domestic capacity development (a question that arose from our interviews), we posed a question of indigeneity–*i*.*e*. how much research is driven by domestic (resident) scientists in a country. Our basis of measuring research activities is publications data, and we make an assumption that *corresponding authors* play a key role in the work reported in a paper. Furthermore, the presence of these researchers in a location will impact effective knowledge transfer, training, mentorship and other activities leading to long-term capacity development. The corresponding author is often fully knowledgeable about the work that is presented in the paper and manages the paper through the peer-review process. She may be the researcher who has done the primary work, or is the senior researcher who has been a central part of the work. Our choice of corresponding author allows for striking a balance in the issue of first and last author contributions, where in some fields, the first author represents the researcher who has done the primary work, whereas in some cases the last author is the main driver of the research.

We compute the indigeneity, λ_i_ of country *i*’s scientific publications, as:

$$\lambda_{i}\left(t\right)=\frac{x_{i}\left(t\right)}{X_{i}\left(t\right)}$$ (4)

where *x*_*i*_ is the number of publications in year *t* where the corresponding author has address in country *i*.

We note that the indigeneity metric represents the domestic side of the knowledge production equation, and does not capture the extended ‘scientific wealth’ of a country via its intellectual diaspora in any given year. It is likely that countries that export high percentages of their educated elites would appear to have less successful educational institutions than those with a less important intellectual diaspora. The metric developed here partially captures the level of domestic scientific capacity and also includes the returning diaspora. It, however, precludes the assessment of export of scientific wealth to other countries as a result of migration out of MENA countries.

### Scientific Specialty–Revealed Scientific Advantage

The disciplinary focus–or specialization in specific scientific fields–is an important characteristic frequently used in assessments of national research [23]. Such characterization is particularly useful in international comparisons where countries with small total shares in global publications may have larger shares in specific areas. In past research, it has been found that emerging countries often show sharp patterns of specialization (with prominent focus in a few fields) whereas scientifically strong as well as scientifically weak countries may show no particular pattern [1].

In this work, we employ the concept of Revealed Comparative Advantage (RCA)—used for determining national trade advantage compared to global exports- in the new context of knowledge economies and apply the definition of RCA on publications (Section A in S1 File). The RCA of publications in different scientific fields has been assessed previously [1], and relative measures for citations are also used that normalize for country size and global activity [23].

Here we denote the RCA for publications as Revealed Scientific Advantage (RSA), and define it as the ratio of the fraction of publications in a subject within a country’s total publications to the fraction of publications in that subject in world total publications. It thus allows for assessing the disciplinary focus of research output of a country while accounting for global trends in research in particular fields. A country with an RSA>1 in a subject area has more publications than the expectation, while an RSA < 1 in a subject indicates publications less than the global level in that area.

An advantage in publications is not a sufficient condition for building strategic capacity, and accounting for quantity of publications is a partial measure at best for gauging strength in research. However, an examination of relative research foci in a country can provide useful information for understanding national trends. Given that research outputs are to some extent organic (and not fully controlled by policy), decision makers and administrators of national science and education agencies can utilize this metric to identify strategic areas for growth.

In our analysis, we agglomerated 175 scientific fields (associated with publications in the Science Citation Index) into 15 disciplines (Table A in S1 File) and evaluated the RSA values in 5-year intervals (Section E in S1 File). We defined Revealed Scientific Advantage (RSA) as:

ρij(t)=xij(t)Xi(t)xWorldj(t)XWorld(t) (5)

where *ρ*_*ij*_*(t)* is the RSA of country *i* in field *j* in year *t*, *X*_i_ is total publications of country *i* in year *t* and *x*_*ij*_ is publications of country *i* in field *j* in year *t*.

### Field-Visits and Interviews

We conducted semi-structured interviews with students, faculty, senior university administrators (including presidents and college deans), technology company executives, and education policy makers in Saudi Arabia, Qatar, Kuwait, and UAE during 2013–2014. In addition, we also made visits to a number of institutions in Morocco, Jordan, Turkey, and Lebanon during 2013–2015. Some of the institutions we visited included: King Abdullah University of Science and Technology (Saudi Arabia), King Fahd University of Petroleum and Minerals (Saudi Arabia), Dhahran Techno Valley (Saudi Arabia), King Abdullah Economic City (Saudi Arabia), Saudi Oil Company (Saudi Arabia), Qatar University, Texas A&M University–Doha (Qatar), Qatar Foundation, Kuwait University, Kuwait Institute for Scientific Research, Kuwait Foundation for Advancement of Science, Advanced Technology Company (Kuwait), Gulf University of Science and Technology (Kuwait), Masdar Institute for Science and Technology (UAE), Abu Dhabi Technology Investment Company (UAE), UAE University- Al Ain (UAE), and Khalifa University (UAE). The on-site interviews and discussions informed the analysis we present here. Some of the key issues that were highlighted included fluctuating (rather than sustained) state support and funding for science, inadequate students’ preparation in science and math in early and secondary level education, negative impacts of socio-political turmoil, and emphasis on international collaborations.

## Results and Discussion

### Global Share and Productivity

**Fig 1:**
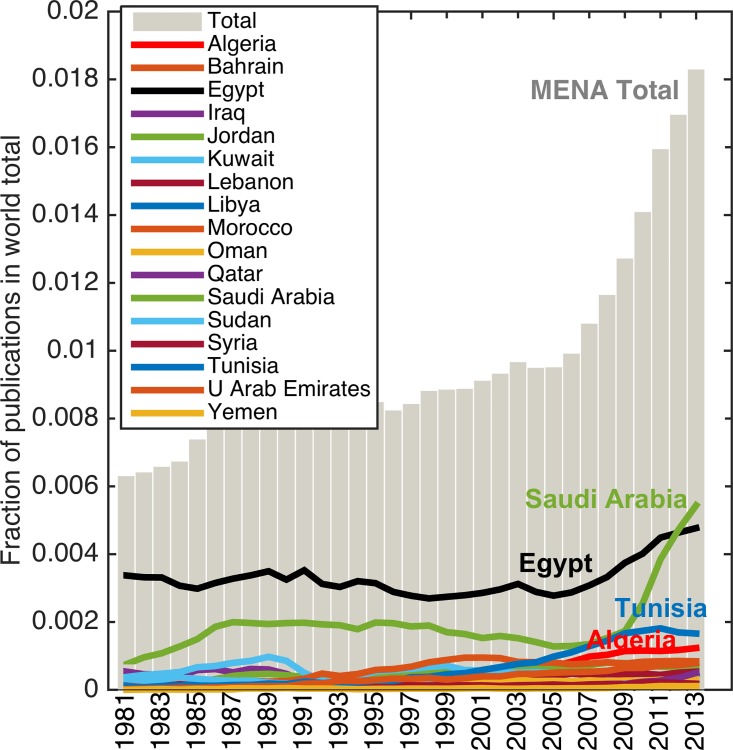
The combined global share in journal publications of all 17 MENA countries increased from 0.63% in 1981 to 1.83% in 2013.

As a first step, we determined the share of total publications of each MENA country over time, and found that a few countries have made gains in the last decade (Table 1). The combined global share for the region grew from ~0.63% in 1981 to 1.83% in 2013 (Fig 1). In 2013, the regional top five countries (in global share) were Saudi Arabia (0.54%), Egypt (0.48%), Tunisia (0.16%), Algeria (0.12%), and Morocco (0.08%). The combined share of Saudi Arabia and Egypt comprised more than half of the total MENA share in global publications. The development trajectory of the two countries, particularly in the last decade however, has interesting features.

**Table 1:** Share of global publications, productivity and indigeneity of publications from MENA and other selected countries.

	Global Share of Publications				Productivity [publications/million people]				Indigeneity	
	1981	1991	2001	2011	1981	1991	2001	2011	2001	2011
Algeria	0.01%	0.02%	0.05%	0.11%	0.00	0.00	0.00	0.00	0.62	0.69
Bahrain	0.00%	0.01%	0.01%	0.01%	0.87	0.59	0.79	0.65	0.85	0.54
Egypt	0.34%	0.35%	0.29%	0.45%	24.59	31.94	35.77	81.85	0.76	0.66
Iraq	0.05%	0.03%	0.01%	0.02%	12.82	7.72	2.94	10.89	0.72	0.65
Jordan	0.01%	0.04%	0.05%	0.07%	20.29	55.29	93.96	159.84	0.81	0.71
Kuwait	0.04%	0.05%	0.06%	0.04%	87.53	130.52	241.85	197.46	0.76	0.69
Lebanon	0.03%	0.01%	0.04%	0.04%	32.50	19.26	92.03	145.34	0.64	0.58
Libya	0.01%	0.01%	0.01%	0.01%	14.97	13.07	9.13	19.66	0.55	0.37
Morocco	0.01%	0.03%	0.09%	0.08%	1.53	6.77	27.53	38.02	0.58	0.58
Oman	0.00%	0.01%	0.03%	0.03%	0.00	24.93	98.70	141.83	0.81	0.54
Qatar	0.00%	0.01%	0.01%	0.03%	44.34	105.13	84.99	206.71	0.83	0.49
Saudi Arabia	0.08%	0.20%	0.15%	0.39%	24.03	61.41	61.51	200.78	0.81	0.52
Sudan	0.03%	0.02%	0.01%	0.02%	7.16	4.51	2.46	7.66	0.41	0.47
Syria	0.00%	0.01%	0.01%	0.02%	0.54	4.06	6.59	14.98	0.63	0.58
Tunisia	0.01%	0.02%	0.06%	0.18%	4.42	15.27	50.96	245.93	0.68	0.79
U Arab Emirates	0.00%	0.02%	0.04%	0.07%	5.50	48.74	106.32	114.96	0.77	0.59
Yemen	0.00%	0.00%	0.00%	0.01%	0.00	1.53	2.33	6.95	0.55	0.35
Turkey	0.07%	0.20%	0.75%	1.42%	5.59	19.07	97.82	281.91	0.91	0.89
Singapore	0.04%	0.14%	0.46%	0.61%	58.83	237.95	938.38	1689.33	0.84	0.65
South Korea	0.07%	0.34%	1.86%	2.95%	5.66	40.93	329.98	858.43	0.82	0.83
China	0.31%	1.32%	3.53%	10.30%	1.05	5.92	23.36	110.99	0.87	0.87
Norway	0.60%	0.55%	0.56%	0.62%	490.28	668.51	1041.71	1809.98	0.71	0.59
USA	40.62%	35.24%	26.11%	20.36%	593.20	723.93	770.85	943.67	0.83	0.79
Australia	2.51%	2.15%	2.26%	2.47%	564.41	646.09	979.76	1599.37	0.77	0.65

Saudi Arabia’s publications share, starting at 0.07% in 1981, initially improved for a few years, after which there were two decades of stagnation and decline. A turning point arrived in 2007 when the number of publications started to increase sharply, a time that coincides with large investments in higher education. The global share of Saudi publications climbed from 0.13% in 2006 to 0.54% in 2013. This rapid increase has garnered attention and debate. A number of experts in our semi-structured interviews noted specific policy measures, new programs for higher education and research, and programs for incentivizing faculty to increase publications as the drivers for this growth (Section A in S1 File). However, some also pointed out the difficulty in clearly gauging the development of local scientific base in the country with publications data given the extensive non-resident, visiting-affiliations for international researchers in recent years (Section B in S1 File). This insight inspired our development of the indigeneity measure for a better assessment of the local knowledge base in countries aiming to develop scientific research capacity.

In Egypt, with a global share of 0.33% in 1981 (the largest share for any single MENA country at the time), there was little growth and mostly stagnation till 2005. The trajectory turned upwards around 2007 in part due to the launching of a ten-year (2007–16) scientific research development plan along with several joint cooperation programs in science and technology with the European Union, Japan, Germany, India, and other countries (Section A in S1 File). There was steady growth until 2011 (coinciding with the Egyptian revolution) after which the rate of increase has slowed (Figure D in Section C in S1 File).

**Fig 2:**
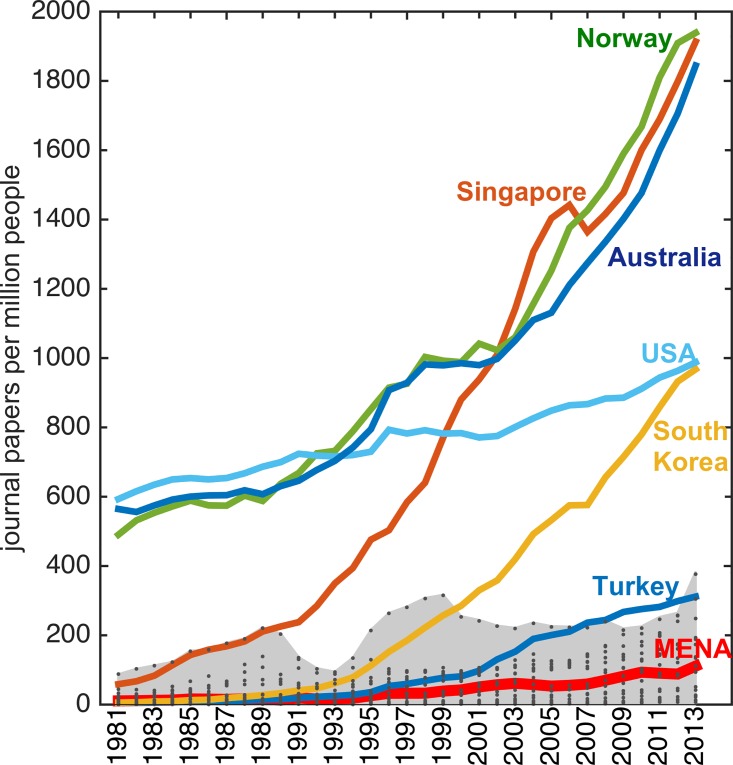
Median productivity (publications/population) in MENA countries (solid red line) shows an enlarged gap in comparison to Singapore, Norway, Australia, and South Korea. The dots show data of individual MENA countries, the grey region encompasses the range of data for MENA.

When compared along an international yardstick, the shares for the comparison group of seven countries ranged from 19.2% for US to 0.6% for Norway in 2013 (Figure B in Section B in S1 File). The total global share of Egypt (largest MENA country with population of 82 million) at 0.48% was lower than the share of Norway (smallest country in our comparison group with population of 5 million) at 0.6%. The productivity results (publications normalized by population) show that in spite of some gains made in terms of total publications, a wide gap has opened up between the productivity of MENA region and some countries in the comparison group (Fig 2). The median productivity in MENA increased by 10.6% on average per year between 1981 and 2013 changing from 11 to 111 publications per million people (with Qatar achieving the highest regional level of 377 papers per million in 2013) (Figure B in Section B in S1 File). In the comparison group, on the other hand, South Korea had an average annual productivity growth rate of 24.7% (from 6 to 968 papers per million), Turkey 18.7% (from 6 to 312 papers per million), and Singapore 16.3% (from 59 to 1913 papers per million) during the same time period (Fig 2). Overall countries in the comparison group progressed faster thereby increasing the productivity gap with MENA region.

**Fig 3:**
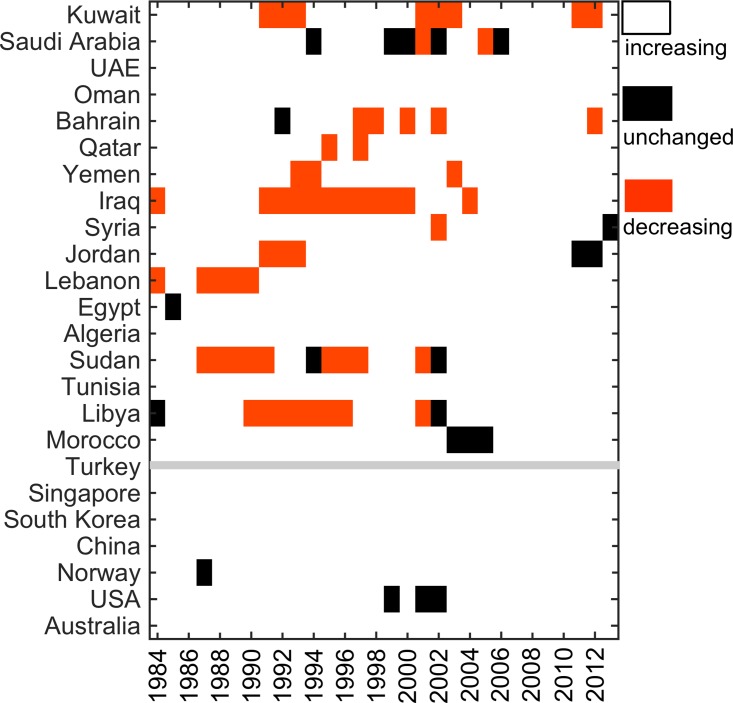
Average rates of change in annual publications show repeated instances of decline and stagnation (shown in red and black) in publications from MENA countries in contrast to countries in the comparison group (below the gray line). The average-three year publications growth rate was computed and classified as unchanged if it was between -0.2% to 0.2%, increasing if greater than 0.2% and decreasing if less than—0.2%.

In digging deeper into the growth trajectories, individual MENA countries show year-to-year fluctuating patterns of stagnation, decline, and growth (Figures B-E in Section C in S1 File). Several countries have repeatedly undergone a decrease in annual output (a contraction in annual number of publications from one year to the next) leading to an erosion of any earlier gains. This partly explains the widened productivity gap. With the exception of Tunisia, Algeria, Egypt, Lebanon, and Oman, all other MENA countries show multiple instances of negative growth (less than -0.2%) or stagnation (growth rate between -0.2% to 0.2% with the results generally robust for other thresholds such as 0.1% or 0.5%) during 1991–2013 (Fig 3).

In Tunisia, productivity increased from 4.4 in 1981 to 248.2 (papers per million) in 2013. However, due to socio-political unrest since 2011, the growth rate has stalled in the country (Figures B-E in Section C in S1 File). Similar impacts of turmoil and military conflicts are visibly perceptible in the data for others including Iraq (1991–1993 and 2003–2004), Kuwait (1991–1993), and Syria (2012–2013).

The rapidly rising trajectories of Singapore and South Korea (as evident in Fig 2) show the impact of sustained policy focus on national technological development (in the backdrop of a more peaceful internal social environment unmarked by military conflict). The rate of growth accelerated in some countries (Qatar, Morocco, Egypt) in MENA in the last seven years (Figures B-E in Section C in S1 File). The crucial issue, however, will be to sustain a rising trend that (at least in the past three decades) has proven to be a tough challenge in the region [24].

### Indigeneity

We conducted the indigeneity analysis for the period 2000–2013. The details of the computation and robustness of the results are provided in Table D in S1 File. For the cases where statistical sampling was used, the margin of error was ~4% or less at a 95% confidence level.

**Fig 4:**
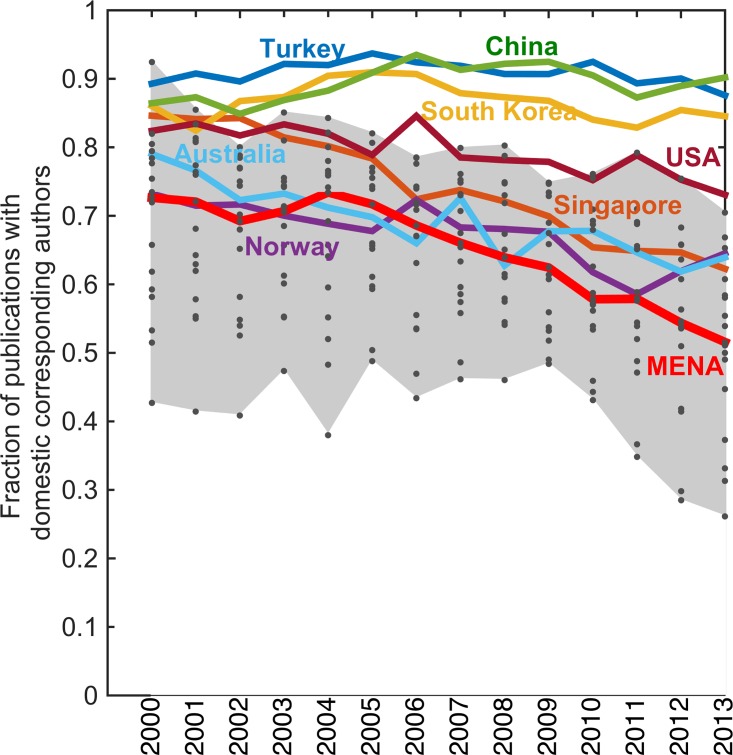
Share of papers with domestic corresponding authors (solid red line shows median value and dots are data of individual countries in MENA). Median indigeneity in 2013 is 52% in MENA compared to 73% in 2000. Algeria had highest (70%) and Yemen had lowest (26%) indigeneity levels in 2013.

The results at the regional level show that the share of local research (as measured by corresponding authors based in the country) has consistently fallen with median for MENA region changing from 73% in 1981 to 52% in 2013 (Fig 4). At the country level, Algeria (70%) had the highest while Yemen (26%) had the lowest indigeneity in 2013 (Figure B in Section D in S1 File). Yemen is followed by Libya at 31% and Qatar at 33% level of domestic corresponding authorship (*i*.*e*. indigeneity).

In Yemen and Libya, the local socio-political unrest and lack of funding support can be attributed to the low levels of domestic research. In the case of Qatar, however, foreign collaborations have been extensively pursued (Section B in S1 File). Senior research administrators in the region pointed out in our interviews that the international collaborations are being pursued for acquiring new knowledge and technical capabilities, and for bringing nascent local programs of teaching and research at par with global practices. In some cases, local researchers seek foreign collaborations due to insufficient local critical mass and lack of local collaborators in their specific fields. The combined effect of these efforts at the macro-level in the country is an indigeneity level that is among the lowest in MENA states.

In the comparison group, China, Turkey, and South Korea have maintained a relatively high degree of indigeneity (from 85–89% in 2000 to 84–90% in 2013), whereas in Australia, Norway and Singapore the indigeneity measure has changed from ~73–79% in 2000 to 62–65% in 2013 (Fig 4). The US fares in the middle going from 82% to 73% in this period.

These results show a noticeable shift in rate of change around the middle of the last decade for many countries (Figure B in Section D in S1 File). In investigating further, we find that the median annual indigeneity change for MENA increases from -0.03% in 2000–2005 period to -2.7% during 2006–2013. On the other hand, the median annual change for comparison countries is -0.7% and -0.8% respectively during these periods (Figures C-D in Section D in S1 File). The indigeneity in MENA has thus decreased at more than three folds the rate (-2.7% versus -0.8%) as compared to the other group of countries during 2006–2013.

These decreasing levels can be explained in part with the role of modern information technologies (IT) in reducing transaction costs and increasing international collaborations. Researchers, however, have documented the limits to benefits that can be gained from collaboration showing that remote collaborations can be prone to problems of coordination [25], and that benefits may accrue only to an extent after which reverse trends may take hold wherein more collaboration impedes productivity [26]. Recent research also shows that while geographically dispersed teams have access to diverse information that can potentially increase novelty, the dispersion can make it difficult to integrate and effectively utilize the information for developing new knowledge [27].

Scientific research and innovation have long been an international enterprise. However, with increased global interactions in this century, scholars in development and innovation theory seek to explicitly investigate the emerging Global Innovation Networks [28], and advance the framework of National Systems of Innovation (NSI) that has received significant interest since the 1990s [29]. Researchers note that as countries seek to catch up, the maturity and nature of their NSIs will impact their role, hierarchy, and influence within the global networks. Thus, even though the global scientific enterprise becomes more connected, the level and quality of domestic human capital and scientific expertise remains vital for linking local research to global research efforts and for utilizing global research for local applications and needs.

**Fig 5:**
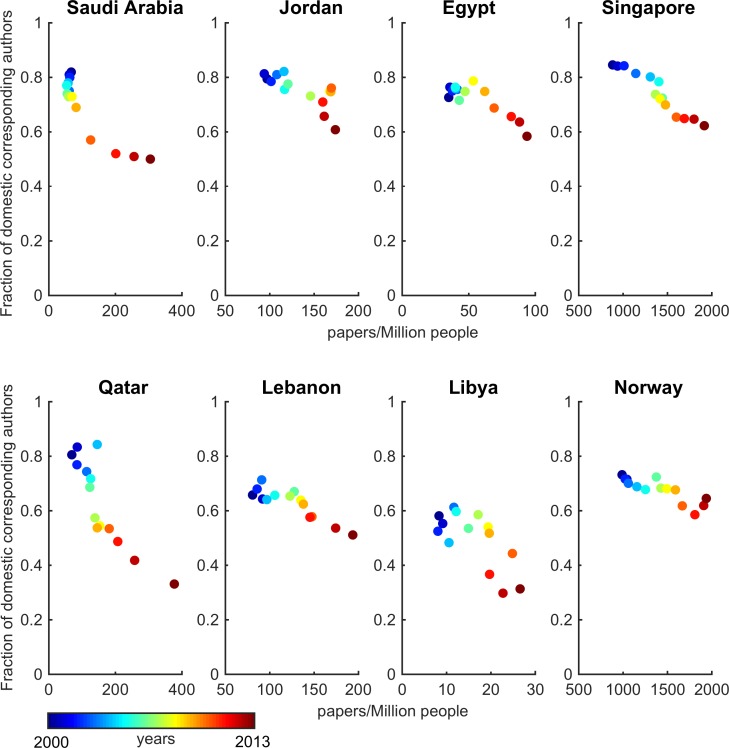
Productivity versus indigeneity shows a negative correlation.

As the question of international collaborations (that are partially captured with the indigeneity metric) is of increasing salience for countries aiming to build local capacity, we analyzed how productivity and indigeneity are related. We found that the correlation between these two metrics was negative (Fig 5), except for a few cases (Figures E-G in Section D in S1 File), showing that the productivity gains in these countries have occurred in step with declining indegeniety. This indicates that international collaborations have contributed to the rise in productivity. The impact of international collaborations on scientific contributions by countries has been noted in previous studies [7, 28], and this result confirms those findings. However, it also provides a further nuance by indicating whether the drivers of the collaborations are domestic or foreign (with country of corresponding author serving as a proxy indicator) and flags implications for countries seeking to develop local capacity.

The overall comparative results show that the locus of scientific work has shifted out of national borders in MENA countries, and raises important questions for domestic scientific research capacity. While other countries (such as Singapore and Norway) also show decreasing trends for indigeneity, the impact of this trend for MENA can be consequential, given the region’s low level of productivity and lack of local research base. The actual (as opposed to expected) impacts of extensive international collaborations needs further understanding as countries in the region attempt to quickly catch up and gain a larger presence in the global research arena.

### Specialty

**Fig 6:**
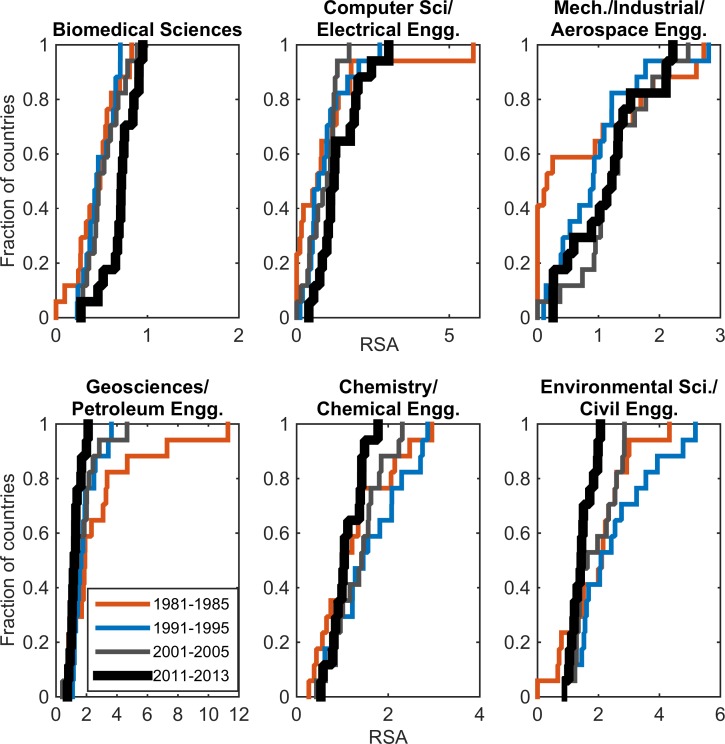
Revealed Scientific Advantage in geological sciences/petroleum engineering, chemistry/chemical engineering, and environmental sciences/civil engineering has waned over time, but has modestly increased in some other areas such as biomedical sciences, computer science/electrical engineering, and mechanical/industrial/aeronautical engineering in the MENA region. Note the different x-scales in each plot.

Our analysis of scientific specialty shows that the focus has shifted from some disciplines such as geological sciences/petroleum engineering to other areas. There is consistent (though modest) growth in the emerging areas of biomedical sciences, in mechanical/industrial/aeronautical engineering, and in computer sciences/electrical engineering (Fig 6). The trends for computer science have to be considered with caution, however, since only journal publications were analyzed, whereas many researchers in this field tend to publish their work in conference papers.

This changing pattern can be attributed to a mix of specific policies such as emphasis on IT and establishment of new regional medical schools in the last decade [30]. The increasing trends in newer areas can have beneficial contributions for scientific competitiveness that can lead to industrial development and economic diversification [31]. However, key questions emerge for the implications of the declining focus on strategically relevant areas (such as in geological sciences and chemical engineering that are relevant to oil and gas sectors) that are expected to remain important for the region in the coming decade.

At the individual country level, patterns of specialization in a few fields are evident (with large RSA values ranging from 4–12 in some subjects) in the first period of the analysis (1981–1985), and the narrow emphasis has broadened over time (Figures A, D-I in Section E in S1 File). In the comparison group, the results show that Norway (with a large oil and gas sector) has maintained strong emphasis in geological sciences/petroleum engineering. Singapore and South Korea (with electronics and semi-conductor industry) have strong focus in materials science (Figures B-C in Section E in S1 File).

This detailed regional and country level characterization of patterns of specialization and research emphasis can serve as an important tool for informing policy and for assessing how research activities may or may not be along trajectories that support broader national development goals and needs.

### Current challenges

In our semi-structured interviews and discussions, which included a wide range of stakeholders ranging from university presidents to faculty and students, a few common issues were raised consistently across all countries we visited: (1) need for “continuity of forward-looking policies” with continued financial support for science and technology over the next couple of decades; (2) “chronic shortage of human capital” and difficulty of attracting good quality faculty in sufficient numbers; (3) insufficient career incentives that have stymied local interest in pursuing long-term research and technical work in science and innovation; and (4) poor quality of school-level education in science and mathematics that leaves students inadequately prepared for university education.

These findings provide explanations for some of the trends and features that emerge from our quantitative bibliometrics analysis. The multiple periods of contraction in scientific output in most MENA countries have left deep uncertainties in academic and research communities. Thus, while they appreciate recent investments, there is persistent concern for long-term sustained support.

In the interviews, several professors pointed to not having a critical mass of other colleagues with whom they have shared interests and complementary areas of expertise in their institution, as well as incentives for faculty that encourage increasing number of publications. The combination of lack of local human capacity and specific measures implemented to boost output may partly explain the rapidly decreasing indigeneity levels that we find in our analysis.

## Conclusions

In this paper we use a combination of classical and new metrics to provide insights into key features of research in countries with relatively poor statistics. We focused on MENA countries for our analysis. It is a region where science once flourished in the early centuries of the last millennium, but a long and persistent drought in scientific inquiry has since prevailed. There are now some signs of change. We find that the share of global publications in science and engineering from MENA has tripled from 0.6% in 1981 to 1.8% in 2013, with the most rapid rise in the last decade (from 2006 onwards) with almost a 1% gain in global share during that time (Fig 1). There is, however, significant variation in progress at country level in the region–and the overall improvements in global share are due to gains in only a few countries.

Furthermore, while increasing output is important, the issue of quality is salient and will ultimately shape the nature of progress in research capabilities and national development. A key limitation of this analysis is that we have not included the usual metrics of publications quality (such as citation counts or impact factors of journals in which papers are published). Some previous studies have assessed the quality of MENA publications on a limited basis [9]. In this work, we sought to quantify the *extent* of activities using a lower bound (total annual publications, regardless of citation counts) for comparison of countries that are in relatively early stages of developing modern research systems.

Our results show that a significant part of the progress in MENA has come about through international collaborations, consistent with (but going beyond) global trends, and that MENA countries are diversifying away from a few areas of scientific focus. This shifting geography of research may be a precursor for catch-up in scientific capabilities in the future, and may prove to be a new model wherein developing countries extensively partner with other nations, in the longer term, to come at par on the international science arena. On the other hand, however, the trends of increasing productivity gap and decreasing indigeneity as compared to other countries (with more established science base) may be symptoms of fragility in the domestic research eco-system in MENA. Recent political unrest and lack of opportunities have accelerated migration in many MENA countries, so the trends may represent a phenomenon that is out of the control of national research organizations. An assessment of the productivity of the diaspora could provide important strategic insights for channels of collaboration and future domestic capacity building.

The measured change from specialty in a few key areas to a more even emphasis across different scientific areas in many MENA countries (Figures D-I in Section E in S1 File) also provides important indication of an evolving system–the direction of which may or may not be aligned with long-term national development goals. The results of our study–and more importantly the approach we have presented- can be used to benchmark and inform strategic decision-making. Here, we have focused on quantitative metrics and trends, however, we explore broader societal, institutional, and policy challenges in [32–33].

Overall, the trends of increased research output are a positive sign. However, other countries are moving faster. Growth in MENA will need to be maintained and further accelerated, as the pressures for expanding economic opportunities for an increasingly young population continue to build where the average annual population growth rate of 3.35% is among the highest in the world (Table B in S1 File).

Regional conflicts, socio-political instability, and lack of sustained support in the past decades for higher education and research have contributed to a widening rather than a narrowing gap in productivity with other countries (Figs 2 and 3). The checkered history of two steps forward and one step back needs to be supplanted with consistent performance–and that can only come about with state-level support in concert with public engagement.

## Supporting Information

S1 File Section A in this file provides further details of the data and methods used in computing metrics using Eqs 1–5. Table A in this file lists the aggregation of 175 subject areas and their classification in Web of Science subject categories. Section B in the file provides details on recent developments in science and technology in MENA. Tables B and C in this section provide data of population and annual publications respectively. Section C has discussion and figures on publications growth rates. Section D has discussion and figures on scientific research indigeneity in MENA countries and the relationship between productivity and indigeneity. Section D also has Table D that summarizes error margins of the statistical sampling in the indigeneity analysis. Section E has additional figures on scientific research areas and Revealed Scientific Advantage of each of the 25 countries studied in this analysis.
(PDF)

## References

[1] May RM (1997) The Scientific Wealth of Nations, Science, 275 (5301): 793–796.

[2] Freeman C (1987) Technology Policy and Economic Performance: Lessons from Japan (Pinter, London).

[3] Solow R (1957) Technical Change and the Aggregate Production Function, Review of Economics and Statistics, 39:312–20.

[4] Schumpeter JA (1934) The Theory of Economic Development (Oxford University Press)

[5] Cohen W M, Levinthal D A (1990) Absorptive Capacity: A New Perspective on Learning and Innovation, Administrative Science Quarterly, 35: 128–152.

[6] Narula R (2003) Understanding Absorptive Capacities in an “Innovation Systems” Context: Consequences for Economic and Employment Growth, Danish Research Unit for Industrial Dynamics Working Paper No. 04–02.

[7] Toivanen H, Suominen A (2015) The Global Inventor Gap: Distribution and Equality of World-Wide Inventive Effort, 1990–2010. PLoS One, 10.1371/journal.pone.0122098 PMC4388728 25849202

[8] Gonzalez-Brambila CN, Reyes-Gonzalez L, Veloso F, Perez-Angon MA (2016) The Scientific Impact of Developing Nations, PLoS ONE 11(3): e0151328 10.1371/journal.pone.0151328 27023182 PMC4811426

[9] Adams J, Christopher K, Pendlebury D, Hook D, Wilsdon J, Zewail A (2011) “Exploring the changing landscape of Arabian, Persian, and Turkish research”, (Global Research Report, Thomson Reuters).

[10] Zewail A (2014), Dire need for a Middle Eastern science spring, Nature Materials, 13:38–320. 10.1038/nmat3918 24651414

[11] King D (2004) The Scientific Impact of Nations: What different countries get for their research spending, Nature, 430: 311–316. 10.1038/430311a 15254529

[12] Hicks D, Wouters P, Waltman L, de Rijcke S, Rafols I, (2015) Bibliometrics: The Leiden Manifesto for research metrics, Nature, 520:429–431. 10.1038/520429a 25903611

[13] Leydesdorff L, Rafols I (2009) A Global Map of Science Based on the ISI Subject Categories, Journal of the American Society for Information Science and Technology, 60 (2): 348–362.

[14] Coile R C (1977) Lotka’s Frequency Distribution of Scientific Productivity, Journal of the American Society for Information Science, 28(6):366–370.

[15] Hicks D, Melkers J (2012) in Handbook on the Theory and Practice of Program Evaluation. Eds. Link Al & Vornatas Nick. Edward Elgar.

[16] Jaffe A B, Trajtenberg M, Henderson R (1993) Geographic localization of knowledge spillovers as evidenced by patent citations. Quarterly Journal of Economics 108: 577–598

[17] Freeman C (1995) The ‘National System of Innovation’ in a historical perspective, Cambridge Journal of Economics, 19:5–24.

[18] Sorenson O, Rivkin J W, Fleming L (2006) Complexity, networks and knowledge flow. Research Policy 35: 994–1017.

[19] McKelvey M, Alm H, Riccaboni M (2003) Does co-location matter for formal knowledge collaboration in the Swedish biotechnology-pharmaceutical sector? Research Policy, 32: 483–501.

[20] Wuchty S, Jones B F, Uzzi B (2007) The Increasing Dominance of Teams in Production of Knowledge, Science, 316: 1036–1039. 10.1126/science.1136099 17431139

[21] Jones B F, Wuchty S, Uzzi B (2008) Multi-University Research Teams: Shifting Impact, Geography, and Stratification in Science, Science, 322:1259–1262. 10.1126/science.1158357 18845711

[22] Adams J, (2013) The fourth age of research, Nature, 497:557–560. 10.1038/497557a 23719446

[23] “National Science Board Science and Engineering Indicators 2014” (2014) NSB 14–01, National Science Foundation.

[24] Zahlan A B (2012) Science, Development, and Sovereignty in the Arab World, (Palgrave Macmillan).

[25] Walsh J P, Maloney N G (2007) Collaboration Structure, Communication Media, and Problems in Scientific Work Teams, Journal of Computer-Mediated Communication, 12:712–732.

[26] Uzzi B, Spiro J (2005) Collaboration and Creativity: The Small World Problem, American Journal of Sociology, 111 (2): 447–504.

[27] Tzabbar D and Vestal A (2015) Bridging the Social Chasm in Geographically Distributed R&D Teams: The Moderating Effects of Relational Strength and Status Asymmetry on the Novelty of Team Innovation, Organization Science, 26 (3): 811–829.

[28] Albuquerque E, Suzigan W, Kruss G, Lee K (Eds) (2015) Developing National Systems of Innovation: University—Industry Interactions in the Global South, (Edward Elgar, UK).

[29] Nelson R. Ed. (1993) National Innovation Systems: A Comparative Analysis. Oxford University Press, New York.

[30] Hajjar D P, Moran G W, Siddiqi A, Richardson J E, Anadon L D, Narayanamurti V (2014) “Prospects for Policy Advances in Science and Technology in the Gulf Arab States: The Role for International Partnerships”, International Journal on Higher Education, 3 (3): 45–57.

[31] Cimini G, Gabrielli A, Labini F (2014) The Scientific Competitiveness of Nations. PLoS One 9(12): e113470 10.1371/journal.pone.0113470 25493626 PMC4262272

[32] Siddiqi A, and Anadon L (Eds) (in press) Science and Technology Development in the Gulf States: Economic Diversification Through Regional Collaboration, (Gerlach Press, Berlin).

[33] Siddiqi A, Anadon L, Narayanamurti V (in press) “Science and Engineering Education in the GCC: Challenges and Transformations” in Higher Education Investment in the Arab States of the Gulf: Strategies for Excellence and Diversity, edited by Eickelman Dale F. and AbuSharaf Rogaia Mustafa (Gerlach Press, Berlin).

