# Supplementary material for: Scientific Wealth in Middle East and North Africa: Productivity, Indigeneity, and Specialty in 1981–2013
Source: PLoS One. 2016 Nov 7;11(11):e0164500. doi: 10.1371/journal.pone.0164500 (PMC5098740; doi:10.1371/journal.pone.0164500)
Supplement: S1 File — Section A in this file provides further details of the data and methods used in computing metrics using Eqs 1–5. Table A in this file lists the aggregation of 175 subject areas and their classification in Web of Science subject categories. Section B in the file provides details on recent developments in science and technology in MENA. Tables B and C in this section provide data of population and annual publications respectively. Section C has discussion and figures on publications growth rates. Section D has discussion and figures on scientific research indigeneity in MENA countries and the relationship between productivity and indigeneity. Section D also has Table D that summarizes error margins of the statistical sampling in the indigeneity analysis. Section E has additional figures on scientific research areas and Revealed Scientific Advantage of each of the 25 countries studied in this analysis. (PDF) [file pone.0164500.s001.pdf]

File S1: Supplementary Materials

Scientific Wealth in Middle East and North Africa: Productivity, Indigeneity, and Specialty in 1981 – 2013

Authors: Afreen Siddiqi[1,2]*, Jonathan Stoppani[3], Laura Diaz Anadon[1,4], Venkatesh Narayanamurti[1,5]

Affiliations:

[1] Belfer Center for Science and International Affairs, John F. Kennedy School of Government, Harvard University, Cambridge, MA, USA.

[2] Institute for Data, Systems, and Society, Massachusetts Institute of Technology, Cambridge, MA, USA.

[3] Haute école spécialisée de Suisse occidentale, Boulevard de Perolles 80, 1700 Fribourg, Switzerland.

[4] Department of Politics and International Studies, University of Cambridge, Cambridge, UK.

[5]School of Engineering and Applied Sciences, Harvard University, Cambridge, MA, USA

*Corresponding Author:

Afreen Siddiqi, Harvard Kennedy School, 124 Mt. Auburn St., Cambridge, MA 02138. Email: afreen_siddiqi@hks.harvard.edu

Keywords:

Middle East science | scientific productivity | bibliometrics | indigeneity | specialty | scientific capacity | knowledge economy | international collaborations | scientific advantage | catch-up| absorptive capacity| national innovation system

Section A: Methods and Data

We used the peer-reviewed published journal paper as the basic unit of scientific output, and conducted our analysis of scientific activity in the MENA countries on data sourced from the Science Citation Index - Expanded™ accessed by Web of Science™ Core Collection that includes over 8500 leading scientific and technical journals. The outputs of scientific activities are much more than published papers, bibliometric analysis nevertheless allows for comparisons and quantitative measurements of the system that are traceable over time. Measuring the quantity of research output is an important part of informed policy making, and is employed by government agencies tasked with assessing national scientific activities *(20).* Citations and impact factors can also provide important insights. However, we focus on quantity of output since our focus of study is countries seeking to develop nascent scientific research activities for which publication volume would be a more useful assessment measure.

We analyzed seventeen countries: Morocco, Libya, Algeria, Tunisia, Sudan, Egypt, Jordan, Lebanon, Syria, Iraq, Yemen, Saudi Arabia, Oman, United Arab Emirates, Bahrain, Kuwait, and Qatar. Arabic is the dominant language in all of these countries, but scientific and technical research is primarily published in English (~98-99%). The known English language publication bias *(23)* will therefore not distort significantly the productivity comparison among the MENA countries, although it will affect the cases of China and some other countries that are provided as context for the MENA data.

Data collection and analysis. Publications data for this study was obtained from the Science Citation Index-Expanded using advanced search queries executed in the online search engine on the Web of Science™ Core Collection. We included full journal articles only (and did not account for letters, reviews, conference papers, books or other publications). The queries were performed during 2014 through 2015. Due to the consistent growth of journals indexed in the database, it is likely that there may be some differences in exact publication counts for queries executed at a later time. The focus of our work was on analyzing total scientific publications in nations that have not had significant research activities in the recent past. Therefore, we chose the Science Citation Index-Expanded (rather than the more widely used Science Citation Index) since it has wider coverage (although of varying journal quality).

The yearly population data was obtained from the Data Bank web portal of the World Bank *(24)* that was used for computing per capita annual publications for each country. Population data used for 2013 is shown in Table B.

Publications Data

We obtained publications volume data for each country using advanced search queries of the form: "CU='<country name>' with publication type specified as 'journal article' and 'English' language. The advanced search field tag, 'CU', searches for countries in addresses fields within records *(25)*. The results of each country's search were counted by year using the Web Of Science analysis tool.

Author Location Data

For author location data, we obtained the full citation records of all publications between 1981-2013 for Kuwait, Qatar, UAE, Saudi Arabia, Bahrain, Oman, Yemen, Sudan, Libya, Syria, and

Iraq. For other countries (with larger number of publications), we obtained random samples of 500 records for each year and analyzed address information of reprint authors in that sample (using our text parsing routines coded in Matlab^{TM}) to statistically estimate indigneity.

Subject Area Data

We obtained subject area data for each country by analyzing the country results in multi- year intervals (1981-85, 1986-90, 1991-95, 1996-2000, 2001-2005, 2006-2010, and 2011-2013) with 'research area' analysis in Web of Science. This provides count of papers for each area. It should be noted that a paper may be assigned multiple subject areas (e.g. it may have two areas Ecology, and Marine & Freshwater Biology) associated with it. We found the world total annual publications in each area by running advanced search queries of the form: "WC=<subject area name>', and then sorted the results by year. We conducted this search for 175 areas (Table A) that we determined to be relevant leaving out areas from social sciences (such as economics, business management *etc*.)

The subject areas were consolidated into 15 categories (Table A) to allow for simpler presentation of results and high-level insights. The categories were based on results reported in *(21)*, wherein a systematic decomposition of a journal-journal citation matrix was used to identify inter-connected disciplines. We used those results with some modifications for engineering disciplines that are relevant for the regional economies in the MENA.

Table A: Classification of Web of Science Subject Categories

Discipline Category	**Subject Areas listed in Web of Science**
Agriculture	AGRICULTURAL ECONOMICS POLICY, AGRICULTURE DAIRY ANIMAL SCIENCE, AGRICULTURE MULTIDISCIPLINARY, AGRONOMY, FOOD SCIENCE TECHNOLOGY, HORTICULTURE, MATERIALS SCIENCE PAPER WOOD, PLANT SCIENCES
Biomedical Sciences	ANATOMY MORPHOLOGY, ANDROLOGY, BIOCHEMICAL RESEARCH METHODS, BIOCHEMISTRY MOLECULAR BIOLOGY, BIOLOGY, BIOPHYSICS, BIOTECHNOLOGY APPLIED MICROBIOLOGY, CELL BIOLOGY, CELL TISSUE ENGINEERING, DEVELOPMENTAL BIOLOGY, ENDOCRINOLOGY METABOLISM, ENGINEERING BIOMEDICAL, GENETICS HEREDITY, INTEGRATIVE COMPLEMENTARY MEDICINE, MATHEMATICAL COMPUTATIONAL BIOLOGY, MEDICAL LABORATORY TECHNOLOGY, MEDICINE RESEARCH EXPERIMENTAL, MICROSCOPY, MULTIDISCIPLINARY SCIENCES, NUTRITION DIETETICS, OBSTETRICS GYNECOLOGY, ONCOLOGY, PATHOLOGY, PHARMACOLOGY PHARMACY, PHYSIOLOGY, REPRODUCTIVE BIOLOGY, TOXICOLOGY, UROLOGY NEPHROLOGY
Chemistry / Chemical Engineering	CHEMISTRY ANALYTICAL, CHEMISTRY APPLIED, CHEMISTRY INORGANIC NUCLEAR, CHEMISTRY MEDICINAL, CHEMISTRY MULTIDISCIPLINARY, CHEMISTRY ORGANIC, CHEMISTRY PHYSICAL, CRYSTALLOGRAPHY, ELECTROCHEMISTRY, ENGINEERING CHEMICAL, POLYMER SCIENCE

Clinical Medicine	ANESTHESIOLOGY, CARDIAC CARDIOVASCULAR SYSTEMS, CRITICAL CARE MEDICINE, DENTISTRY ORAL SURGERY MEDICINE, DERMATOLOGY, EMERGENCY MEDICINE, GASTROENTEROLOGY HEPATOLOGY, GERONTOLOGY, HEMATOLOGY, ORTHOPEDICS, OTORHINOLARYNGOLOGY, PEDIATRICS, PERIPHERAL VASCULAR DISEASE, RADIOLOGY NUCLEAR MEDICINE MEDICAL IMAGING, RESPIRATORY SYSTEM, RHEUMATOLOGY, SPORT SCIENCES, SURGERY, TRANSPLANTATION
Computer Science/ Electrical Engineering	AUTOMATION CONTROL SYSTEMS, COMPUTER SCIENCE ARTIFICIAL INTELLIGENCE, COMPUTER SCIENCE CYBERNETICS, COMPUTER SCIENCE HARDWARE ARCHITECTURE, COMPUTER SCIENCE INFORMATION SYSTEMS, COMPUTER SCIENCE INTERDISCIPLINARY APPLICATIONS, COMPUTER SCIENCE SOFTWARE ENGINEERING, COMPUTER SCIENCE THEORY METHODS, ENGINEERING ELECTRICAL ELECTRONIC, INSTRUMENTS INSTRUMENTATION, TELECOMMUNICATIONS
Ecology	BIODIVERSITY CONSERVATION, ECOLOGY, ENTOMOLOGY, EVOLUTIONARY BIOLOGY, FISHERIES, MARINE FRESHWATER BIOLOGY, ORNITHOLOGY, ZOOLOGY
Environmental Science /Civil Engineering	AGRICULTURAL ENGINEERING, CONSTRUCTION BUILDING TECHNOLOGY, ENGINEERING CIVIL, ENGINEERING ENVIRONMENTAL, ENGINEERING GEOLOGICAL, ENGINEERING OCEAN, ENVIRONMENTAL SCIENCES, ENVIRONMENTAL STUDIES, TRANSPORTATION, TRANSPORTATION SCIENCE TECHNOLOGY, WATER RESOURCES
General Medicine/ Health	AUDIOLOGY SPEECH LANGUAGE PATHOLOGY, HEALTH CARE SCIENCES SERVICES, MEDICAL ETHICS, MEDICAL INFORMATICS, MEDICINE GENERAL INTERNAL, NURSING, PRIMARY HEALTH CARE, PUBLIC ENVIRONMENTAL OCCUPATIONAL HEALTH
Geosciences/ Petroleum Engineering	ENERGY FUELS, ENGINEERING PETROLEUM, GEOCHEMISTRY GEOPHYSICS, GEOGRAPHY PHYSICAL GEOLOGY, GEOSCIENCES MULTIDISCIPLINARY, IMAGING SCIENCE PHOTOGRAPHIC TECHNOLOGY, LIMNOLOGY, METEOROLOGY ATMOSPHERIC SCIENCES, MINERALOGY, MINING MINEARL PROCESSING, OCEANOGRAPHY, PALEONTOLOGY, REMOTE SENSING, SOIL SCIENCE
Infectious Diseases	ALLERGY, IMMUNOLOGY, INFECTIOUS DISEASES, MICROBIOLOGY, MYCOLOGY, PARASITOLOGY, TROPICAL MEDICINE, VETERINARY SCIENCES, VIROLOGY
Materials science	MATERIALS SCIENCE BIOMATERIALS, MATERIALS SCIENCE CERAMICS, MATERIALS SCIENCE CHARACTERIZATION TESTING, MATERIALS SCIENCE COATINGS FILMS, MATERIALS SCIENCE MULTIDISCIPLINARY, MATERIALS SCIENCE TEXTILES, METALLURGY METALLURGICAL ENGINEERING, NANOSCIENCE NANOTECHNOLOGY, PHYSICS APPLIED
Mathematics	LOGIC, MATHEMATICS, MATHEMATICS APPLIED, MATHEMATICS INTERDISCIPLINARY APPLICATIONS, STATISTICS PROBABILITY

Mechanical, Industrial, Aeronautical Engineering	ACOUSTICS, ENGINEERING AEROSPACE, ENGINEERING INDUSTRIAL, ENGINEERING MANUFACTURING, ENGINEERING MARINE, ENGINEERING MECHANICAL, ENGINEERING MULTIDISCIPLINARY, MATERIALS SCIENCE COMPOSITES, MECHANICS, ROBOTICS, THERMODYNAMICS
Neurosciences	BEHAVIORAL SCIENCES, CLINICAL NEUROLOGY, GERIATRICS GERONTOLOGY, NEUROIMAGING, NEUROSCIENCES, OPTHALMOLOGY, PSYCHIATRY, PSYCHOLOGY, REHABILITATION, SUBSTANCE ABUSE
Physics/Nuclear Sciences	ASTRONOMY ASTROPHYSICS, NUCLEAR SCIENCE TECHNOLOGY, OPTICS, PHYSICS ATOMIC MOLECULAR CHEMICAL, PHYSICS CONDENSED MATTER, PHYSICS FLUIDS, PLASMAS, PHYSICS MATHEMATICAL, PHYSICS MULTIDISCIPLINARY, PHYSICS NUCLEAR, PHYSICS PARTICLES FIELDS, SPECTROSCOPY

Field-Visits and Interviews. We conducted semi-structured interviews with students, faculty, senior university administrators (including presidents and college deans), technology company executives, and education policy makers in Saudi Arabia, Qatar, Kuwait, and UAE during 2013-2014. In addition, we also made visits to a number of institutions in Morocco, Jordan, Turkey, and Lebanon during 2013-2015. Some of the institutions we visited included: King Abdullah University of Science and Technology (Saudi Arabia), King Fahd University of Petroleum and Minerals (Saudi Arabia), Dhahran Techno Valley (Saudi Arabia), King Abdullah Economic City (Saudi Arabia), Saudi Oil Company (Saudi Arabia), Qatar University, Texas A&M University – Doha (Qatar), Qatar Foundation, Kuwait University, Kuwait Institute for Scientific Research, Kuwait Foundation for Advancement of Science, Advanced Technology Company (Kuwait), Gulf University of Science and Technology (Kuwait), Masdar Institute for Science and Technology (UAE), Abu Dhabi Technology Investment Company (UAE), UAE University- Al Ain, Khalifa University (UAE). The on-site interviews and discussions informed the analysis we present here. Some of the key issues that were highlighted included shifting (rather than sustained) state support and funding for science, inadequate students' preparation in science and math in early and high-school education, negative impacts of socio-political turmoil, and emphasis on international collaborations.

Definitions and Equations

1. Publications Volume

The total publications for a country *i* in year *t* was defined as:

$$X_i(t) = \#\text{ of publications with atleast one author address in country i} \quad (1)$$

The whole-counting approach was used, where for instance, if a publication had three co-authors, and one of the co-authors had an address in Kuwait, the publication would be included as a full count for Kuwait. This approach provided an upper limit accounting of the publications for each

country. The attribution for each country was made only the basis of address information and the citizenship or national origin of authors was not taken into account.

The global share of each country was computed for each year *t* as:

$$\text{Share of country i in world publications } (t) = \frac{X_i(t)}{\sum_{i=1}^{N} X_i(t)} \qquad (2)$$

where *N* is the number of countries with journal publications records in year *t*.

2**. Scientific Productivity**
The productivity was measured as the ratio of annual publications and population for each country. We computed the scientific productivity, $|_i$ of country *i* in year *t*, as:

$$\eta_i(t) = \frac{X_i(t)}{P_i(t)} \qquad (3)$$

where $P_i(t)$ is population of country *i* in year *t*.

This ratio (of total publications to total population) has been used in past work *(1,2)*. Ideally, the number of total scientists and researchers should be used instead of total population of a country. The productivity measures computed for US and OECD countries typically use data of scientific research workforce. This data however is not available for many countries where science and technology sectors are not well developed. In such cases, the total population serves as a proxy variable for determining productivity.

The productivity values computed with total population numbers have to be treated with caution, since the demographics in MENA countries are heavily skewed towards younger ages. The 0-14 years age group constitutes 28% of the population on average in the region (Table B). In the selected countries used for comparison, the 0-14 years age group constitutes 19% of the population on average (Table B).

3. **Scientific Indigeneity**
In this measure we are interested in assessing the extent of the scientific output that can be attributed to researchers resident in a country. Co-location of researchers is an important issue, since it impacts the speed and type of knowledge transfer, and efficiency and quality of collaborations driven by shared research interests. Furthermore, in some cases it has been found that geographic proximity is important for university-firm interactions *(14)* – and this can have important implications for workforce training, as well as innovation, and industrial competitiveness. The impact of geography on knowledge transfer is an active area of research given the new globalizing trends, increased mobility and ease of communications.

We use the country addresses of corresponding authors to account for scientific output for each country. The corresponding author is often fully knowledgeable about the work that is presented in the paper and manages the paper through the peer-review process. She may be the researcher

who has done the primary work, or is the senior researcher who has been a central part of the work. Our choice of corresponding author allows for striking a balance in the issue of first and last author contributions, where in some fields, the first author represents the researcher who has done the primary work, whereas in some cases the last author is the main driver of the research. We compute the indigeneity, ⌊i of country *i*'s scientific publications, as

$$\lambda_i(t) = \frac{x_i(t)}{X_i(t)} \qquad (4)$$

where x_i is the number of publications in year t where the corresponding author has address in country i.

We recognize that there are limitations with this approach. The contact addresses of the corresponding author may not accurately reflect the location where the published work was actually conducted. Furthermore, researchers frequently move, or often have multiple concurrent affiliations with institutions located in different countries. Nonetheless, the author country addresses provide a verifiable and quantifiable measure for assessing general patterns of location of scientific activities and collaborations, and we utilize this information to conduct our analysis. There is also no reason to believe that this introduces significant biases in the cross-country comparison.

4. **Scientific Specialization – Revealed Scientific Advantage (RSA)**

We analyzed subject areas of publications for each country and compared share of publications in particular subject areas of a country in total world publications. Using the Revealed Comparative Advantage (RCA) concept from international trade theory *(26)*, we defined the Revealed Scientific Advantage (〉$_{ij}$) for a country i in subject j as:

$$\rho_{ij}(t) = \frac{x_{ij}(t) / X_i(t)}{x_{\mathrm{World}\,j}(t) / X_{World}(t)} \qquad (5)$$

The Revealed Scientific Advantage (RSA) is the RCA of a country's publications. It is computed as the fraction of publications in subject j within country i's total publications normalized by the fraction of publications in subject j in total world publications. RSA gives a measure of how the publications output in a subject differs from the world average. Past research has focused on relative citation impact and publications RCA of countries within different fields of science *(1)*. It was found that scientifically strong countries (such as the US and Japan) and scientifically weak countries (such as Papua New Guinea) had no particular pattern of specialization. To the best of the knowledge of the authors, there has been no recent assessments of field specific specializations for MENA region or an updated analysis for the comparison group.

Section B: Recent developments in Science and Technology in MENA

Over the last several decades, Arab countries in the Middle East and North Africa (MENA) region have lagged behind in scientific research *(5)* with insufficient government support and lack of long-term focus on building local capacity in science and technology. However, in recent years the oil-dependent economies of Saudi Arabia, United Arab Emirates, and Qatar in particular have sought to expand their scientific and technological capacity for economic diversification *(27, 28)*. In 2011, Saudi Arabia was among the top 40 countries in the world for R&D spending *(29)*, and in 2014, 56 billion US dollars representing 24% of the total Saudi national budget was allocated for education and training *(30)*. The United Arab Emirates (UAE) has established new universities (such as the Masdar Institute of Science and Technology) and campuses (including NYU-Abu Dhabi, INSEAD-Abu Dhabi and several others), and has sought to become a research hub of renewable energy technologies. The Qatari government established Education City in Doha that includes branch campuses of US engineering and medical schools and established the Qatar National Research Foundation to initiate and build a local research enterprise. Additionally, Egypt launched major efforts to revitalize national science and technology. The Higher Education Reform Strategy (2002 –2017) was introduced to improve the quality of education, the Decade for Science and Technology 2007-16 was announced in 2006, a National Strategic Plan for Pre-University Education Reform was introduced in 2007, several new research funding and training programs and collaborations with other countries (including Japan, Germany) were formally established *(34)*.

Many regional universities – that had historically only focused on teaching and professional training – are embracing research as a key part of their core mission. This adoption of the Humboldtian model in the region, wherein universities are not only repositories of knowledge, but contribute to creating new knowledge (27, 31), is evident through expansion of research faculty in science and engineering departments, creation of new and expanded laboratories, and established of science and technology parks in the region. Middle Eastern universities are actively targeting foreign researchers to relocate to the region to jumpstart and expand local research *(32)*. These efforts – from increased funding to attracting scientific research talent – are bearing some results as publications in MENA countries have been on the upward trajectory (Fig. A).

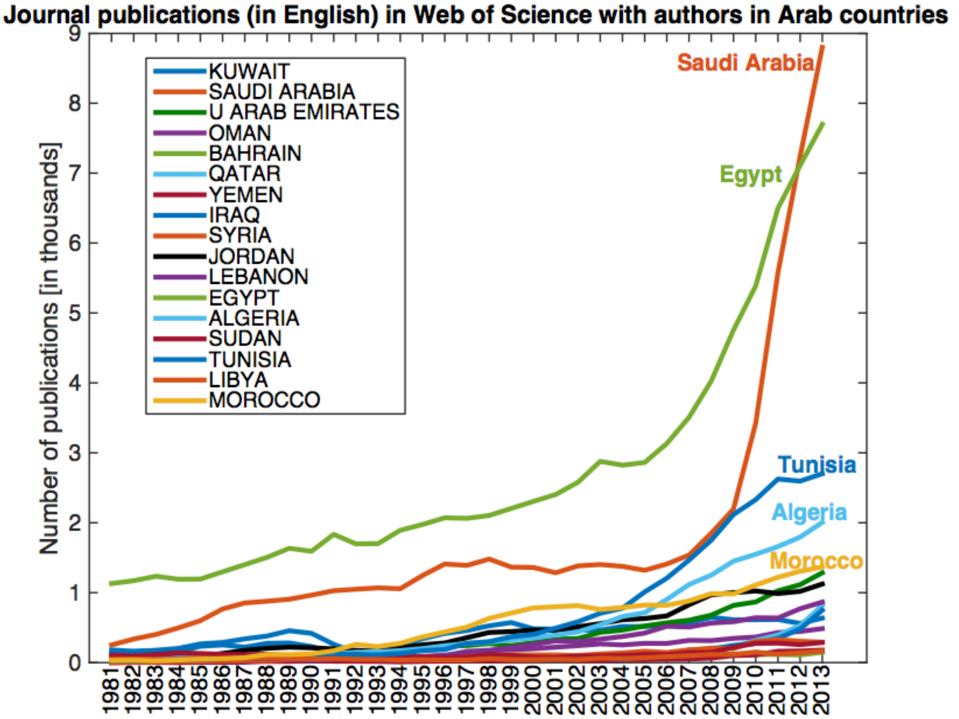

Fig. A: Annual publications in scientific journals with authors from 17 MENA countries. Egypt was the historical leader in total output in the region, but has been surpassed by Saudi Arabia.

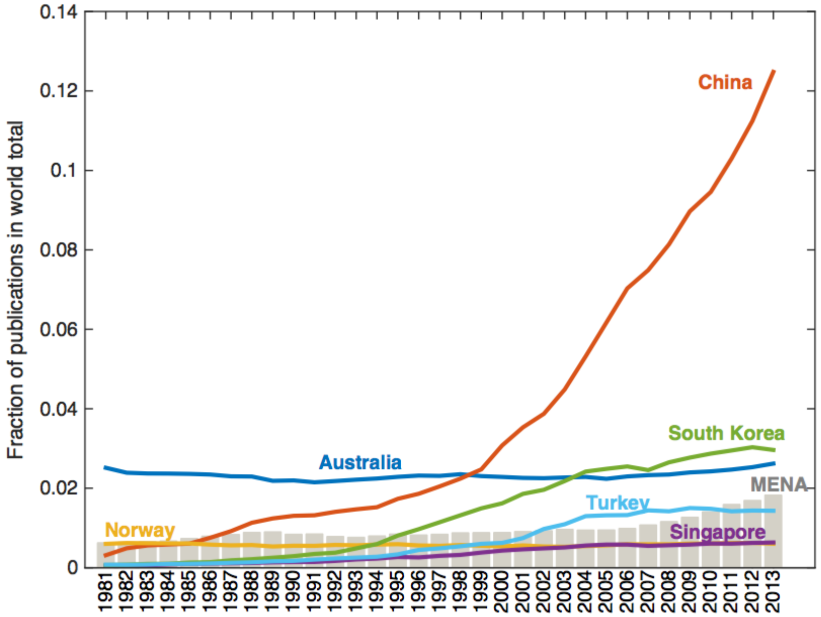

Fig. B: Share of countries in comparison group. China's share has risen from 0.3% in 1981 to 12.5% in 2013. South Korea, Turkey, and Singapore have gained share. Share for US (not shown) decreases from 40.6% in 1981 to 19.2% in 2013.

The top five MENA countries in 2013 were Saudi Arabia, Egypt, Tunisia, Algeria and Morocco in terms of share of global journal publications with 0.54%, 0.48%, 0.16%, 0.12%, and 0.08% respectively. The shares for the comparison group of seven countries in 2013 were: Norway 0.6%, Singapore 0.63%, Turkey 1.43%, Australia 2.61% and South Korea 2.96%, China 12.48% and US 19.2%. It can be noted that the largest MENA countries (Egypt and Saudi Arabia in

terms of publications share) had smaller shares than the smallest countries in the comparison group (Singapore and Norway).

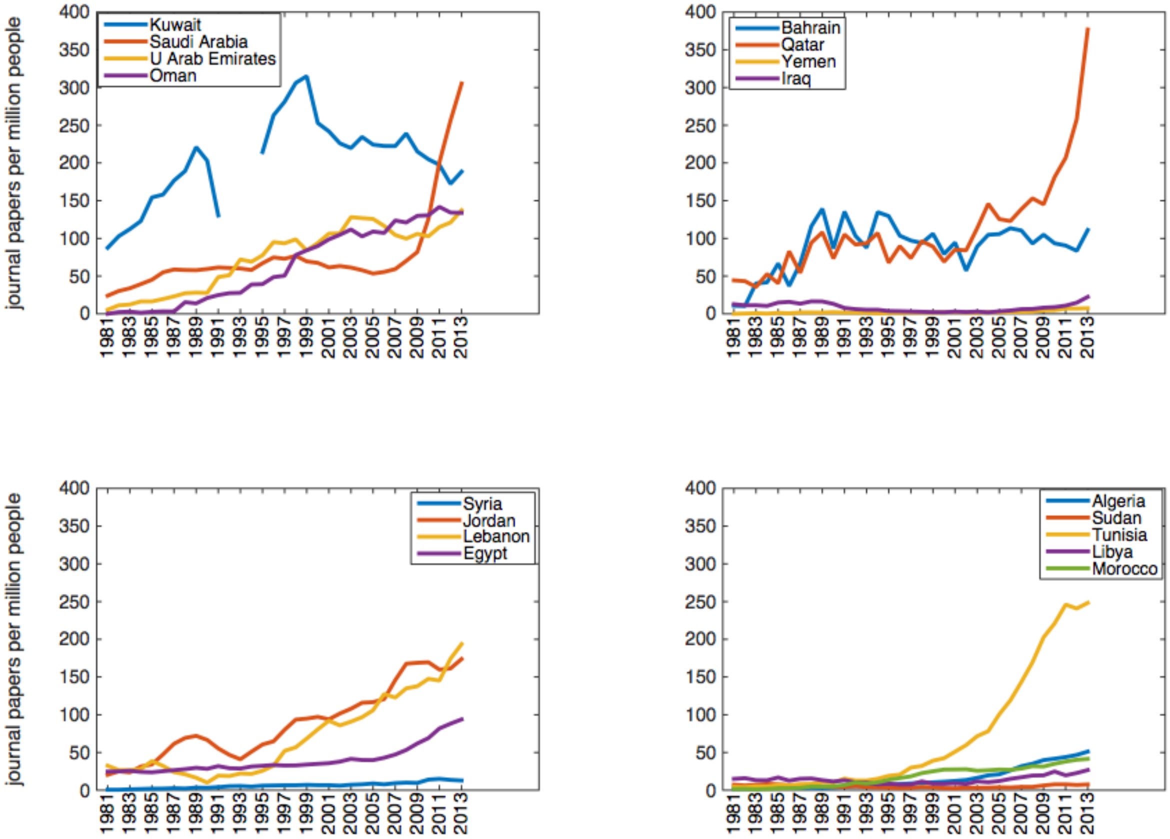

Fig. C: Journal publications per million people in MENA countries between 1981-2013. Data is missing for Kuwait for 1990-91 due to lack of population data for those years.

Table B: Population Data for MENA and comparator group *(33)*

Middle East and North Africa	Total Population (estimated for 2013)	Average annual population growth [%] (2000-13)	Population composition of ages 0-14 years [%]
Algeria	39,208,194	2	28
Bahrain	1,332,171	5	21
Egypt	82,056,378	2	31
Iraq	33,417,476	3	40
Jordan	6,459,000	2	34
Kuwait	3,368,572	4	25
Lebanon	4,467,390	2	21
Libya	6,201,521	1	29
Morocco	33,008,150	1	28

Oman	3,632,444	4	23
Qatar	2,168,673	10	14
Saudi Arabia	28,828,870	3	29
Sudan	37,964,306	2	41
Syria	22,845,550	3	35
Tunisia	10,886,500	1	23
United Arab Emirates	9,346,129	9	15
Yemen	24,407,381	3	40
MENA Total	349,598,381		
MENA Average		3.35	28.05
**Comparator group**			
Australia	23,130,900	1	19
China	1,357,380,000	1	18
Korea, Rep.	50,219,669	1	15
Norway	5,084,190	1	19
Singapore	5,399,200	2	16
Turkey	74,932,641	1	26
United States	316,128,839	1	20
Comparator average		1.14	19

Table C. Annual journal publications from MENA and other selected countries.

	1981	1982	1983	1984	1985	1986	1987	1988	1989	1990
Algeria	40	46	43	63	64	61	66	84	69	96
Bahrain	0	0	16	17	28	16	30	54	67	43
Egypt	1130	1171	1234	1193	1195	1299	1401	1505	1634	1591
Iraq	180	162	169	159	233	252	220	275	280	230
Jordan	46	59	58	80	90	130	175	204	220	211
Kuwait	126	155	177	203	267	289	339	380	455	418
Lebanon	85	70	70	75	103	86	64	56	44	27

Libya	48	53	46	47	63	49	60	63	55	49
Morocco	31	33	24	37	50	58	70	117	114	125
Oman	0	0	0	0	0	0	0	26	24	38
Qatar	11	12	11	18	15	33	23	42	50	35
Saudi Arabia	252	338	402	496	599	766	853	878	906	964
Sudan	107	96	114	139	131	118	114	120	99	89
Syria	0	0	11	15	20	23	29	26	41	39
Tunisia	29	28	36	35	50	49	61	65	86	82
U Arab Emirates	6	13	15	21	22	28	35	44	48	50
Yemen	0	0	0	0	0	0	0	0	0	0
Turkey	251	257	291	360	384	440	522	609	745	835
Singapore	149	178	224	311	392	433	467	520	618	687
South Korea	219	263	345	384	517	574	781	951	1168	1414
China	1047	1720	2085	2266	2427	3108	3928	5039	5787	6391
Norway	2010	2188	2286	2368	2444	2397	2404	2540	2484	2703
USA	136120	142657	148409	153261	155547	156117	158436	163279	169453	174661
Australia	8425	8437	8836	9198	9461	9670	9830	10226	10208	10760

	1991	1992	1989	1994	1995	1996	1997	1998	1999	2000
Algeria	123	149	156	171	201	242	271	292	325	354
Bahrain	69	54	47	74	73	60	58	58	68	53
Egypt	1833	1697	1701	1893	1975	2070	2065	2104	2205	2305
Iraq	139	115	103	105	77	72	67	60	53	51
Jordan	196	175	160	207	254	280	357	430	444	466
Kuwait	261	143	159	239	340	417	460	527	573	482
Lebanon	53	53	64	64	78	100	161	177	216	261
Libya	57	45	45	36	41	36	37	58	43	43
Morocco	170	260	229	276	368	439	507	633	709	783
Oman	47	54	57	82	85	106	110	169	183	197

Qatar	51	45	46	53	34	46	39	53	51	41
Saudi Arabia	1028	1049	1068	1056	1246	1409	1391	1480	1366	1361
Sudan	94	116	93	87	79	71	81	114	102	77
Syria	52	69	76	66	86	92	99	100	113	109
Tunisia	127	109	111	133	170	187	276	300	369	405
U Arab Emirates	93	103	153	154	182	235	244	272	243	284
Yemen	0	6	18	10	18	21	28	28	27	35
Turkey	1047	1256	1416	1623	2106	3188	3606	4207	4815	5146
Singapore	747	920	1159	1350	1678	1847	2218	2515	3049	3546
South Korea	1788	2050	2718	3513	5082	6944	8510	10291	11997	13402
China	6849	7637	8244	8998	10890	13341	15238	17499	19900	25429
Norway	2850	3108	3157	3423	3717	4009	4079	4446	4429	4438
USA	183159	184302	186195	189581	194345	213700	213324	218434	218187	221080
Australia	11173	11849	12428	13252	14363	16617	17197	18371	18530	18870

	2001	2002	2003	2004	2005	2006	2007	2008	2009	2010
Algeria	394	439	540	664	714	902	1117	1250	1450	1550
Bahrain	66	42	69	86	93	108	114	104	125	117
Egypt	2404	2580	2878	2824	2862	3134	3508	4028	4754	5390
Iraq	72	62	78	51	90	123	178	189	247	273
Jordan	462	512	557	613	631	668	826	970	999	1025
Kuwait	479	463	465	515	515	538	568	646	613	614
Lebanon	309	302	335	373	422	521	508	563	586	641
Libya	48	43	63	58	68	84	99	113	116	150
Morocco	799	814	759	796	824	821	881	986	983	1110
Oman	221	242	267	253	274	274	318	314	346	366
Qatar	52	53	75	105	103	119	160	208	227	317

Saudi Arabia	1285	1383	1402	1377	1319	1409	1537	1850	2197	3424
Sudan	70	93	95	100	118	113	142	146	218	276
Syria	110	100	124	137	162	144	182	204	202	301
Tunisia	493	584	705	791	1010	1206	1466	1752	2116	2337
U Arab Emirates	333	344	432	464	522	568	606	678	818	868
Yemen	42	31	29	42	41	49	53	63	101	109
Turkey	6270	8488	10076	12675	13589	14442	16435	17180	19058	19886
Singapore	3883	4218	4702	5447	5987	6346	6262	6854	7366	8135
South Korea	15627	17074	20099	23638	25625	27810	27991	32121	35168	38526
China	29708	33727	41354	51993	63610	76753	85345	98571	113852	126935
Norway	4702	4642	4840	5309	5790	6413	6719	7128	7680	8153
USA	219686	222986	232442	241890	250670	257638	261224	268601	271717	282072
Australia	19019	19611	20911	22338	23062	25061	26540	28389	30418	32540

	**2011**	**2012**	**2013**
Algeria	1660	1804	2022
Bahrain	117	110	151
Egypt	6501	7123	7826
Iraq	347	481	757
Jordan	988	1023	1128
Kuwait	618	560	642
Lebanon	638	774	877
Libya	120	141	167
Morocco	1220	1311	1378
Oman	430	446	489
Qatar	396	536	818
Saudi Arabia	5578	7249	8928
Sudan	279	256	291
Syria	329	309	292

Tunisia	2627	2602	2723
U Arab Emirates	1029	1120	1292
Yemen	162	165	181
Turkey	20565	22084	23488
Singapore	8764	9580	10394
South Korea	42734	46618	48613
China	149198	172829	204790
Norway	8967	9588	9881
USA	294902	303918	315082
Australia	35778	38863	42970

Section C: Publications Growth Rates in MENA Countries and Selected Comparison Countries

We computed rates of change in productivity using a 3-year moving average for 1991-2013. We only included data from 1991 onwards since science activity was small in many MENA countries in the 1980s decade. For year *y*, the average of the growth in *y-2*, *y-1,* and *y* was computed. For 1991, we used data for 1989, 1990, and 1991. The descriptive statistics for the last two decades are shown in Fig. A.

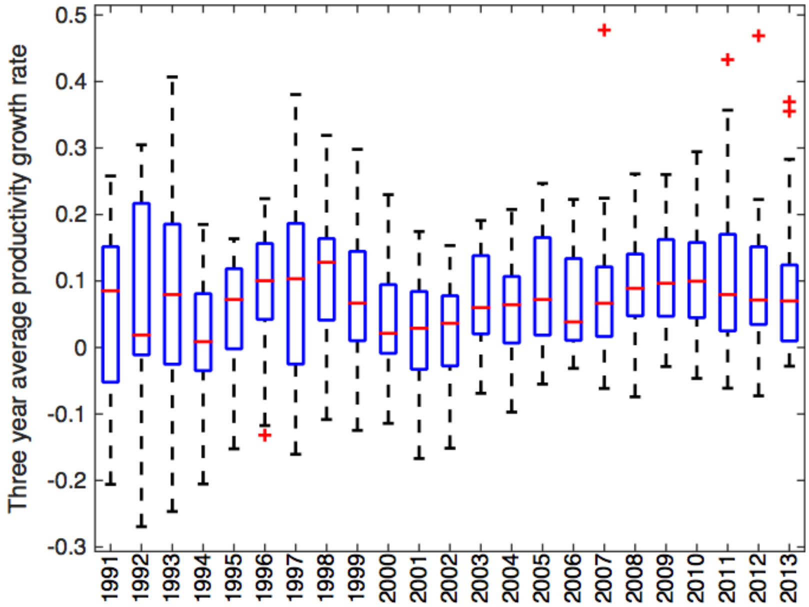

Fig. A. For 17 countries in MENA, median productivity growth rate has been in the range of 1.8% to 12.8% during 1991-2000, between 2.9 – 9.6% during 2001-2010, and 2011-2013 has been 7.9, 7.1, and 6.9%. The outliers in the years 2011-2013 are for Saudi Arabia and Iraq.

The results of individual countries show wide ranging variation with repeating cycles of periods of decline, stagnation, and growth for countries in the Arabian peninsula (Fig. B and C). Since 2007, Saudi Arabia and Qatar have shown sustained growth starting from approximately zero in 2007 to 35% and 28% respectively in 2013. This sustained growth led Saudi Arabia, in 2012, to surpass Egypt and Tunisia– the long time regionally scientific dominant nations - in total publications volume (see Figure A in Section B). There has been, however, debate regarding the rapid increase in publications from Saudi Arabia *(35)*. And some experts in the discussions noted the difficulty of using publications data to assess research in the country wherein there are extensive programs for enlisting international researchers for visiting-affiliations at Saudi institutions. The countries in the Levant (Jordan, Syria, Lebanon), and North Africa also show cycles of growth and decline, with effects of socio-political turmoil and military conflict prominent for Iraq (with a negative productivity growth rate from 1991 to 2000), declining growth for Tunisia and Egypt since 2011, a sharp decline for Syria in 2012 and so on.

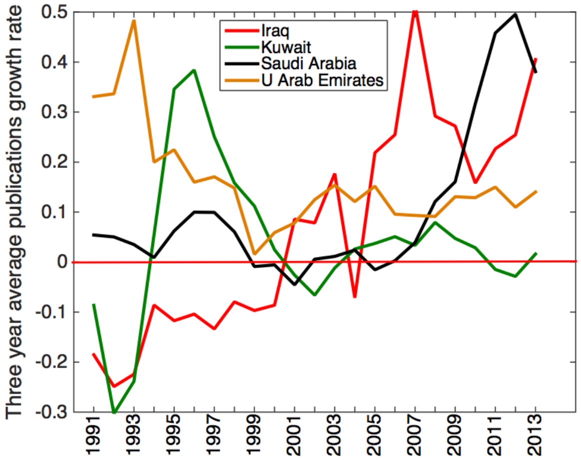

Fig. B. 3-year average rate of change in scientific productivity for Iraq, Kuwait, Saudi Arabia and UAE.

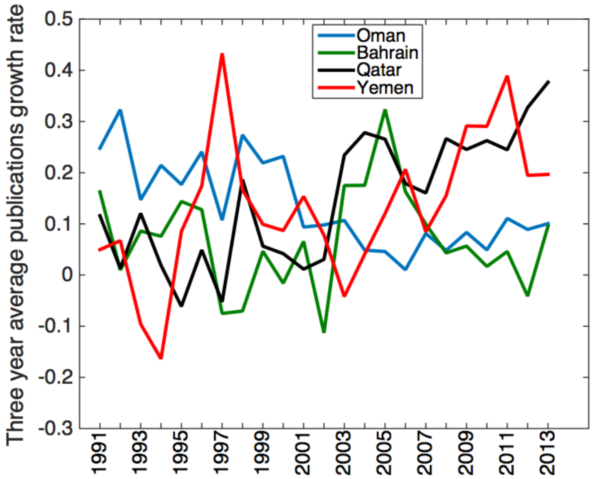

Fig. C. 3-year average rate of change in scientific productivity for Oman, Bahrain, Qatar, and Yemen.

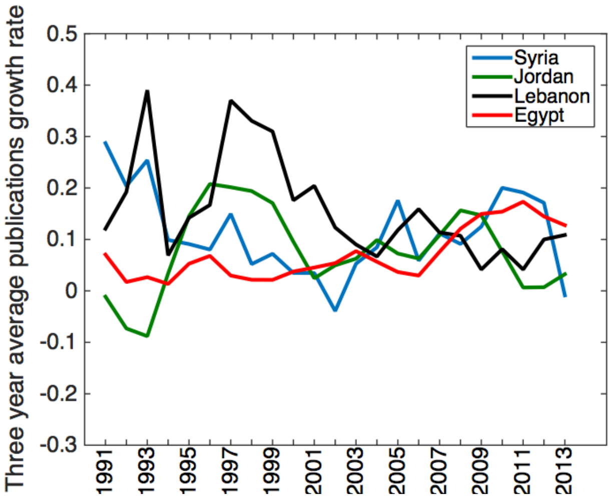

Fig. D. 3-year average rate of change in scientific productivity for Egypt and Levant

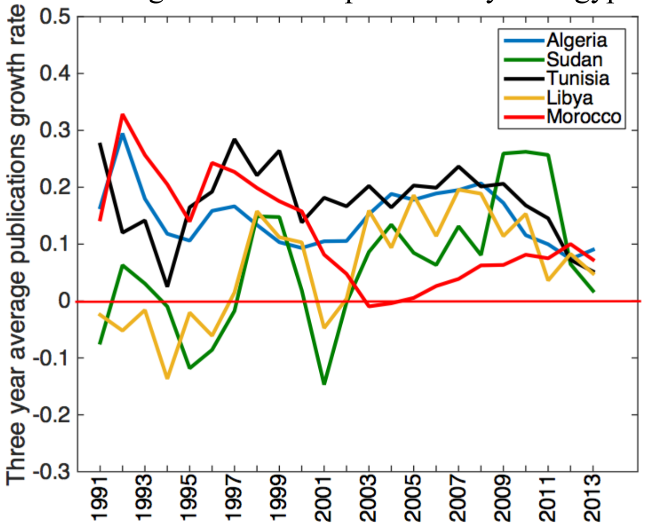

Fig. E. 3-year average rate of change in scientific productivity for countries in North Africa

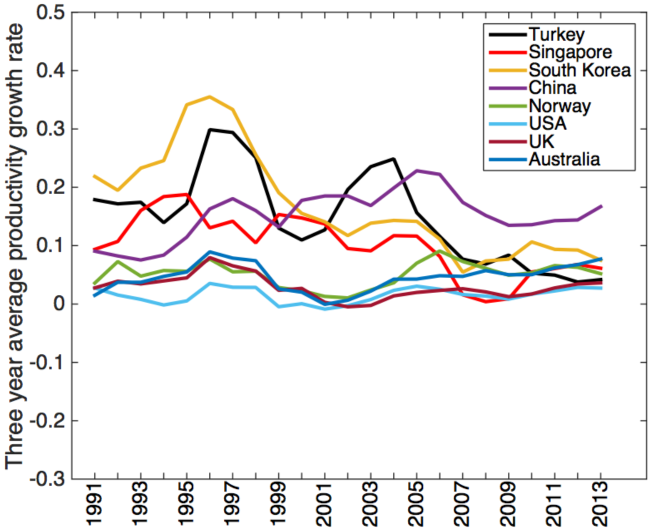

Fig. F. 3-year average rate of change in productivity for selected comparison countries

In comparison with selected countries, it can be noted that while there are cyclical patterns, the growth rates do not fall significantly below zero (which is the case when there is contraction of

annual publications). Figures B-E show that in every year between 1991-2013, the minimum values for the region's 3-year average growth rate has been consistently below zero. This explains why the productivity gap has widened with MENA – while the countries shown above have continued to expand scientific output (though with varying rates) year after year, several countries in MENA have repeatedly undergone a decrease in year to year per capita output leading to an erosion of any gains that may have been made in earlier years. Figures B - E show that 11 out of 17 countries had negative growth in multiple years during 1991-2013. The countries that did not experience contraction are Egypt, Tunisia, Algeria, Lebanon, UAE, and Oman.

Section D: Scientific research indigeneity in MENA Countries

Table D: Indigeneity Computation Error Analysis

	Sample Size	Max Error Margin [%]
Algeria	500	3.8
Bahrain	Full	
Egypt	1000	2.9
Iraq	Full	
Jordan	500	3.3
Kuwait	Full	
Lebanon	500	2.9
Libya	Full	
Morocco	500	3.5
Oman	Full	
Qatar	Full	
Saudi Arabia	Full	
Sudan	Full	
Syria	Full	
Tunisia	500	3.96
UAE	Full	
Yemen	Full	

Turkey	1000	3.05
Singapore	1000	2.95
South Korea	1000	3.07
Norway	1000	4.3
China	1000	3.1
USA	1000	3.1
Australia	500	4.4

The indigeneity was computed for each country for years between 2000 and 2013. A full data set was used for eleven cases (shown above), while 500 or 1000 samples were used for the rest. In cases where total publications for a particular year were less than the sample size (e.g. less than 500 or 1000), then the total number of publications was used. The margin of error in indigeneity computation for each year was equal to or less than the maximum margin of error value (at 95% confidence level) shown for each country in Table D.

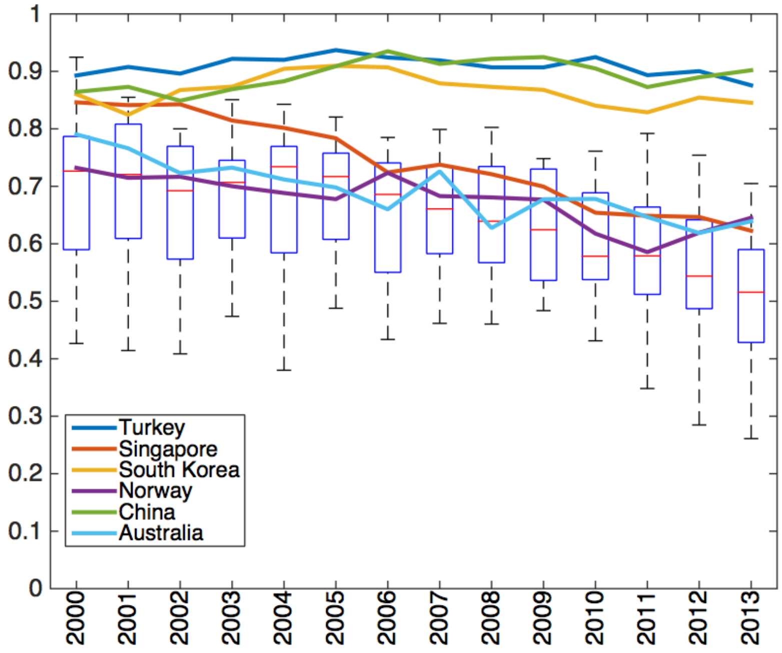

Fig. A. y-axis is fraction of publications with domestic corresponding authors. Box plots showing max, min, median, and inter-quartile ranges of annual indigeneity of scientific research publications from MENA countries. Selected countries from other regions are included for comparison.

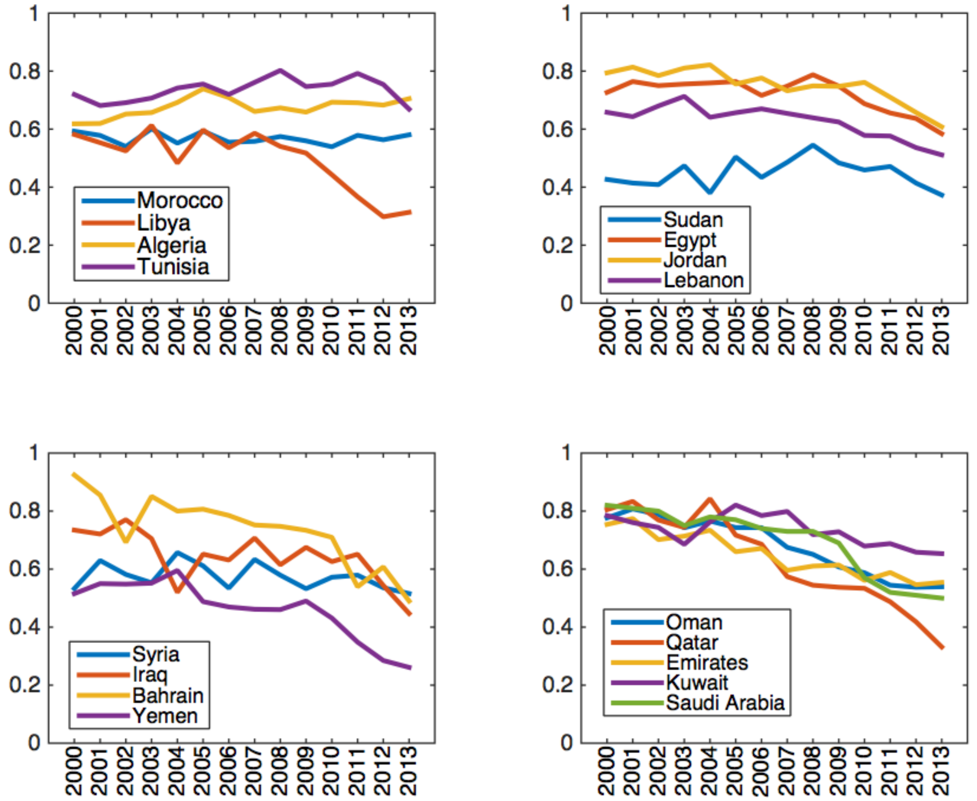

Fig. B: Indigeneity (Fraction of domestic corresponding authors in annual publications) for 17 MENA countries.

An analysis of the rate of change of indigeneity per year finds that since 2006, the median decrease in MENA countries was -2.7% as compared to -0.03% in 2000-2005 period (Fig. C and D in this section). This partly explains the rise in productivity in some MENA countries around that time. The increased volume of publications from these countries is partly due to rapid increase in collaboration driven by international researchers. The median annual change for other countries was -0.7% during 2000-2005 period and -0.8% during 2006-2013 (Fig. C and D in this section).

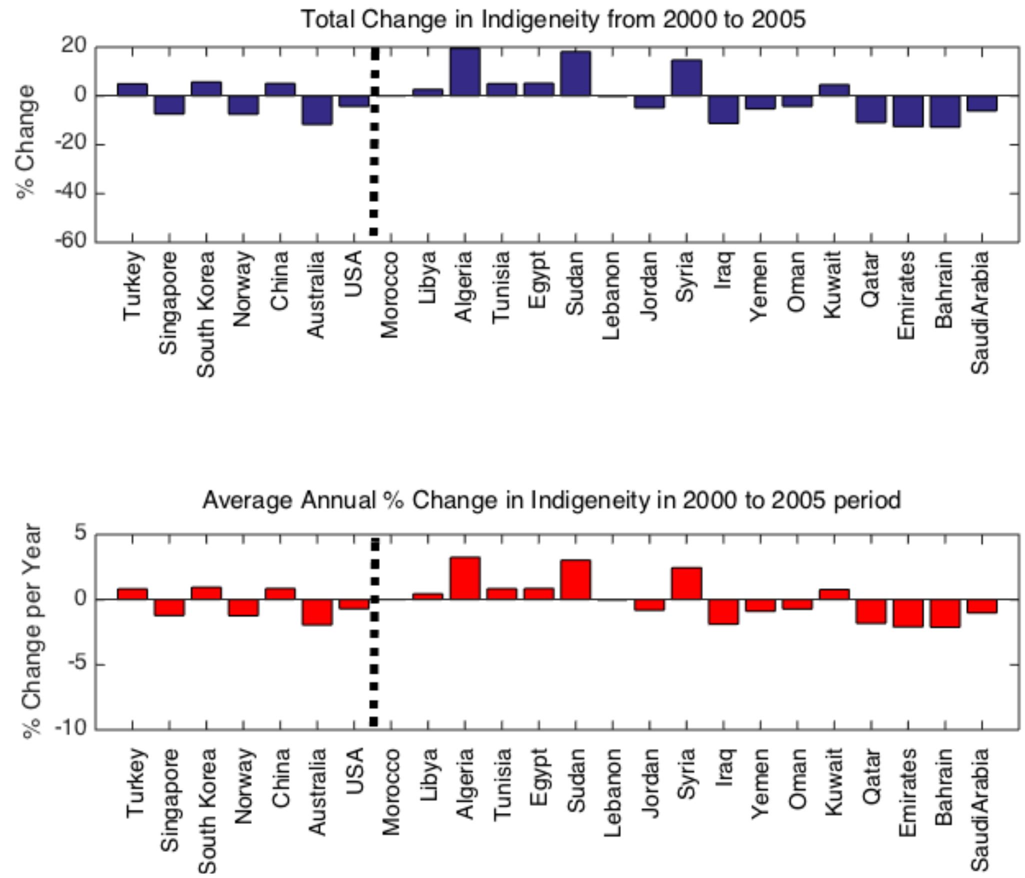

Fig. C: Total percentage change (top) and annual percentage change (bottom) in indigeniety during 2000 - 2005. Median change in indigeneity in MENA is -0.03% per year and -0.7% per year in other group during this period.

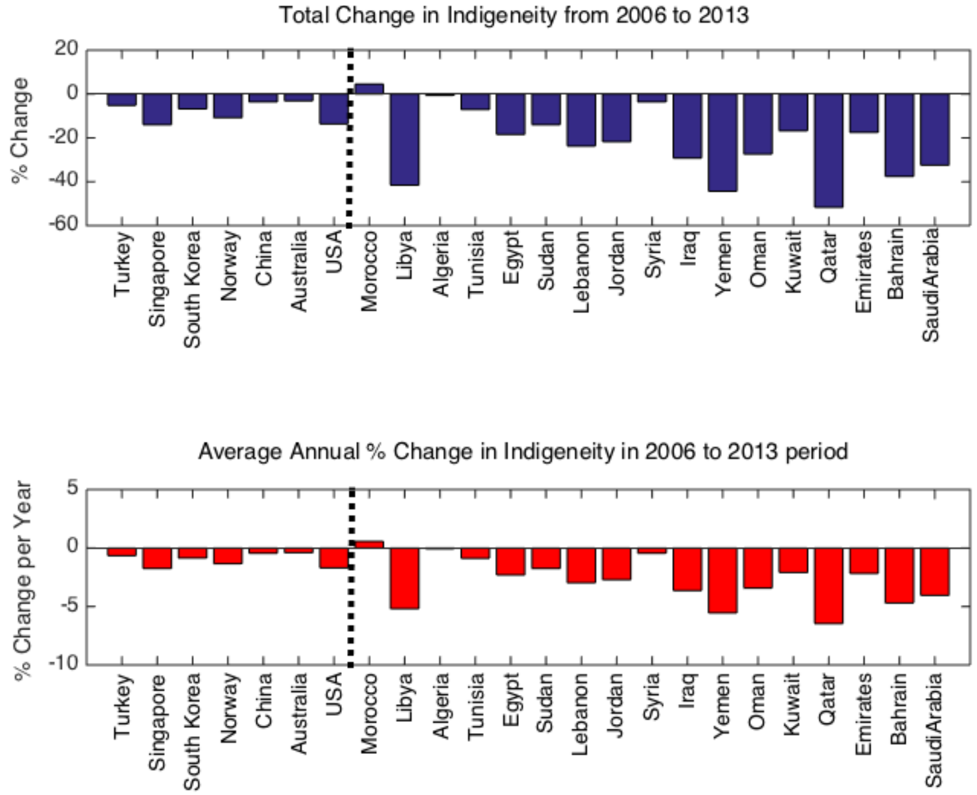

Fig. D: Total percentage change (top) and annual percentage change (bottom) in indigeniety during 2006 - 2013. Median change in indigeneity in MENA is -2.7% per year and -0.8% per year in other group during this period.

When the relationship between a country's productivity and indigeneity was analyzed, we found that in most cases there was a negative linear trend, however Saudi Arabia, Qatar, and United Arab Emirates showed almost exponential decline in indegenity with increasing productivity over time (Fig. E – G in this section). The productivity increases in these countries have occurred in step with decreasing level of indegeneity. In some cases, there were exceptions such as for Turkey, South Korea and China where the negative trend is negligible indicating that productivity gains have been made through expansion of local capacity (wherein fraction of domestic corresponding authors serves as a proxy indicator).

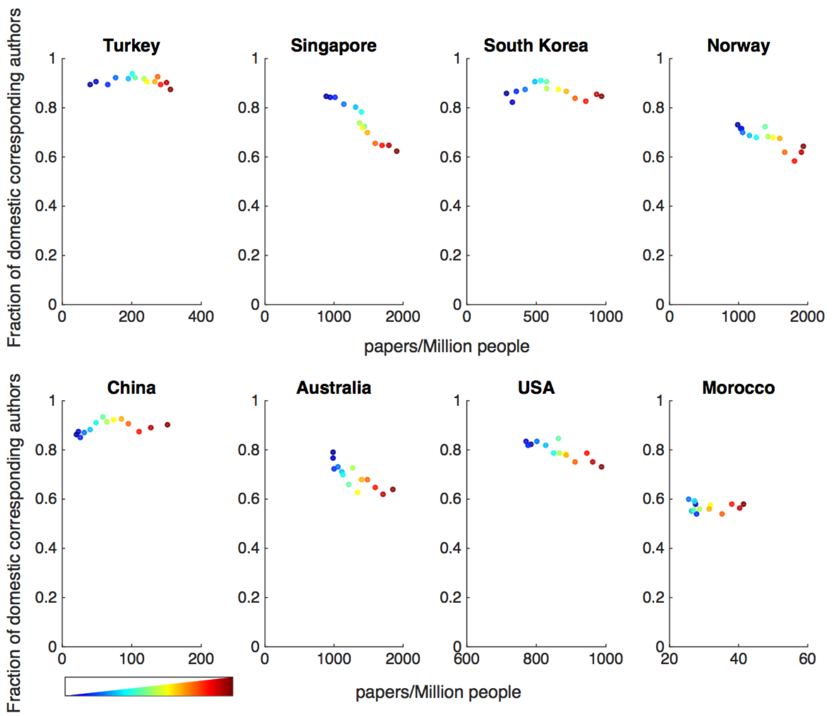

Fig. E. Productivity versus Indigeneity of countries.

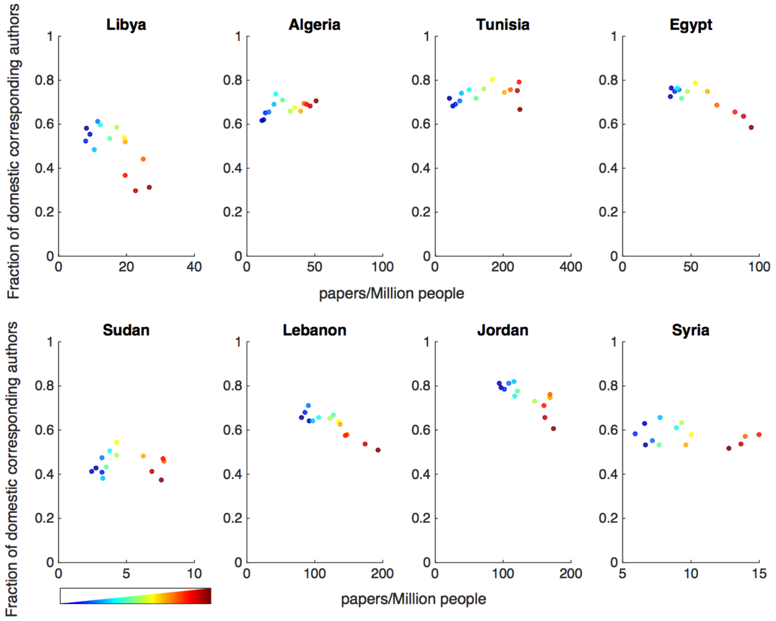

Fig. F: Productivity versus Indigeneity of countries

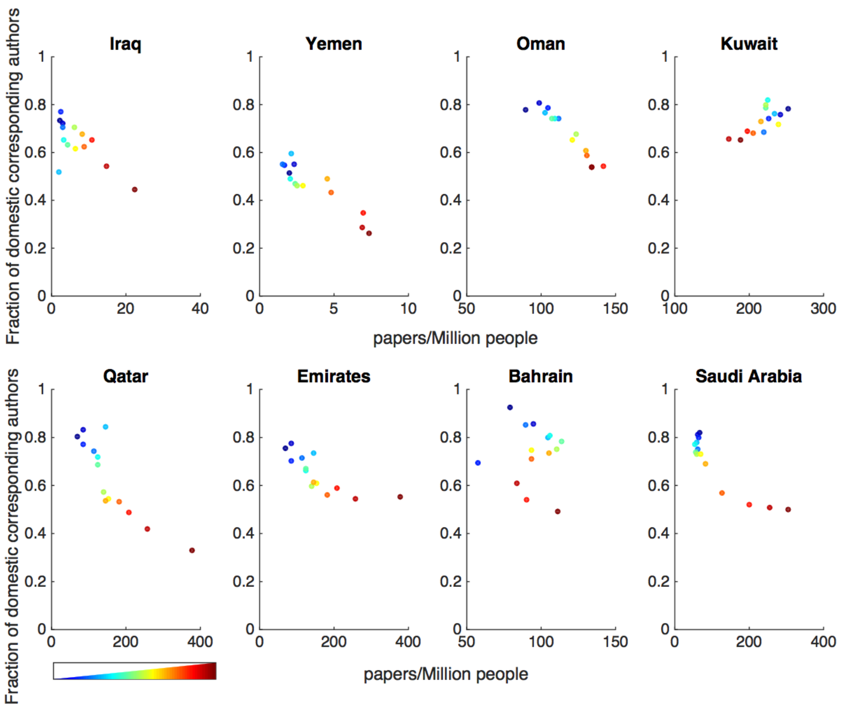

Fig. G: Productivity versus Indigeneity of countries

Section E: Scientific research areas in MENA Countries

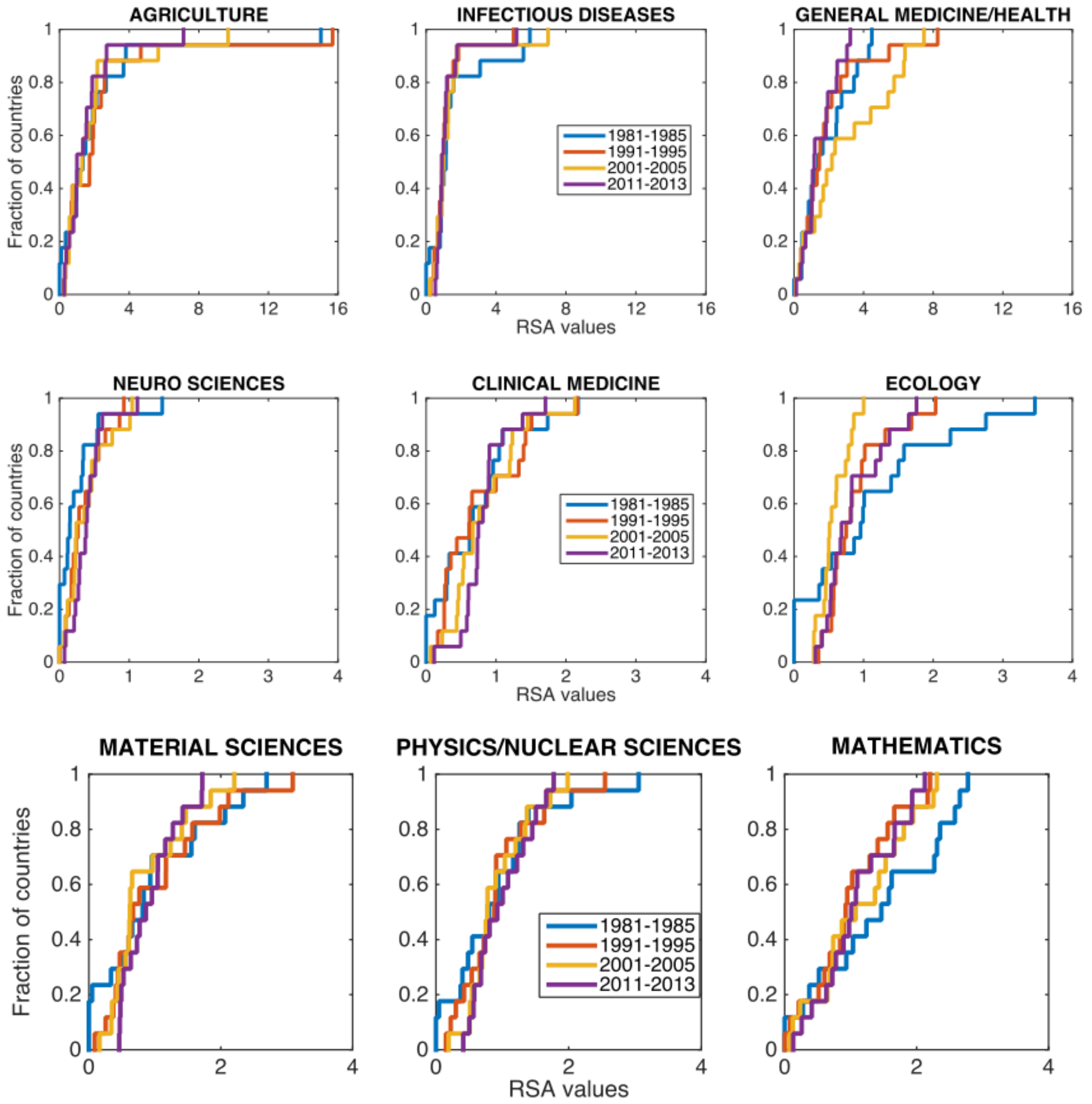

Fig. A. Cumulative distribution of Revealed Scientific Advantage for MENA in subjects with increasing focus (top row) and mixed focus (bottom row). X-scale is different in top row.

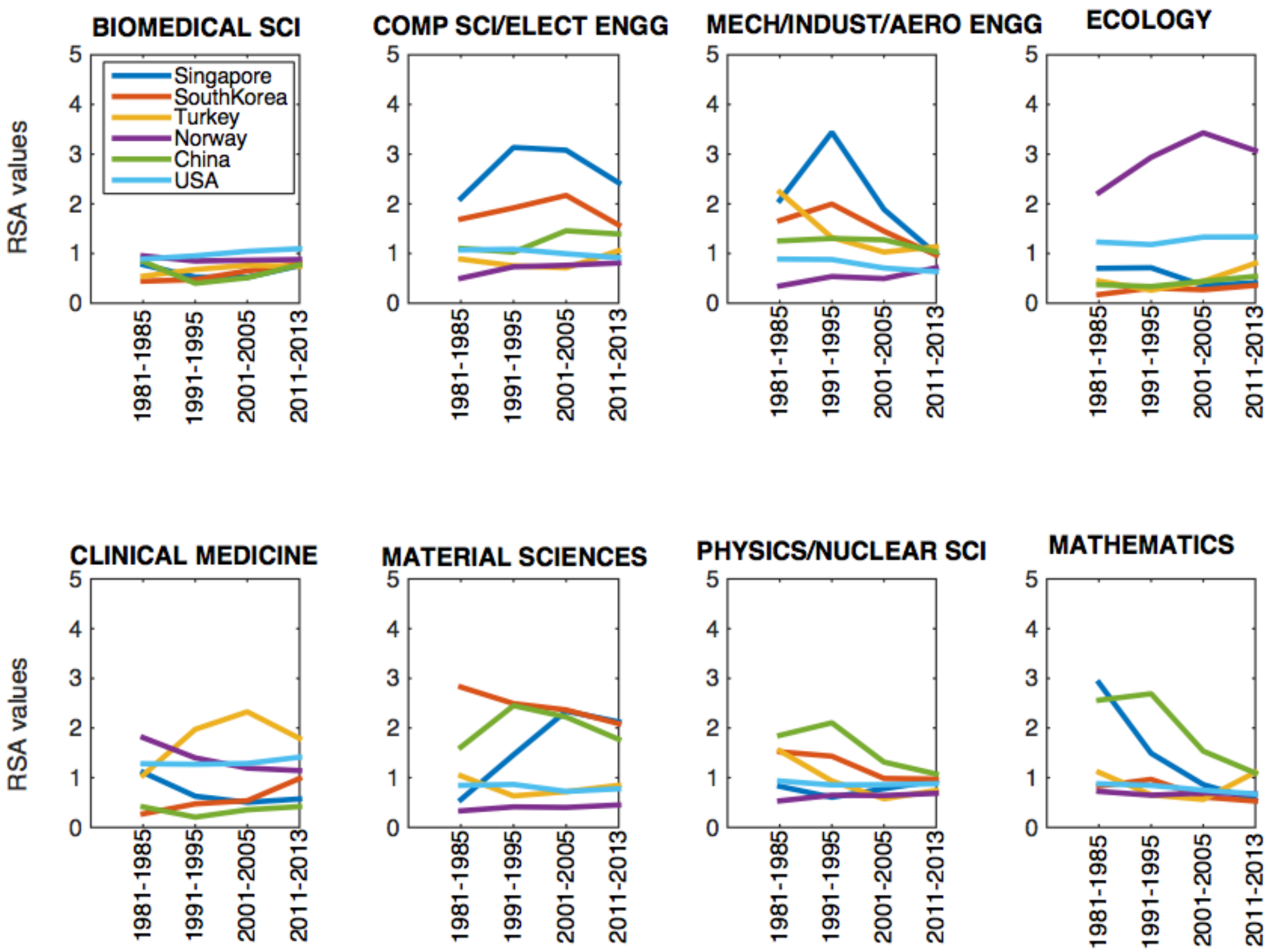

Fig. B. RSA for countries in comparison group. Some patterns that stand out include high RSA in computer science/electrical engineering for Singapore, in ecology for Norway, clinical medicine for Turkey, and in materials sciences for South Korea, Singapore, and China.

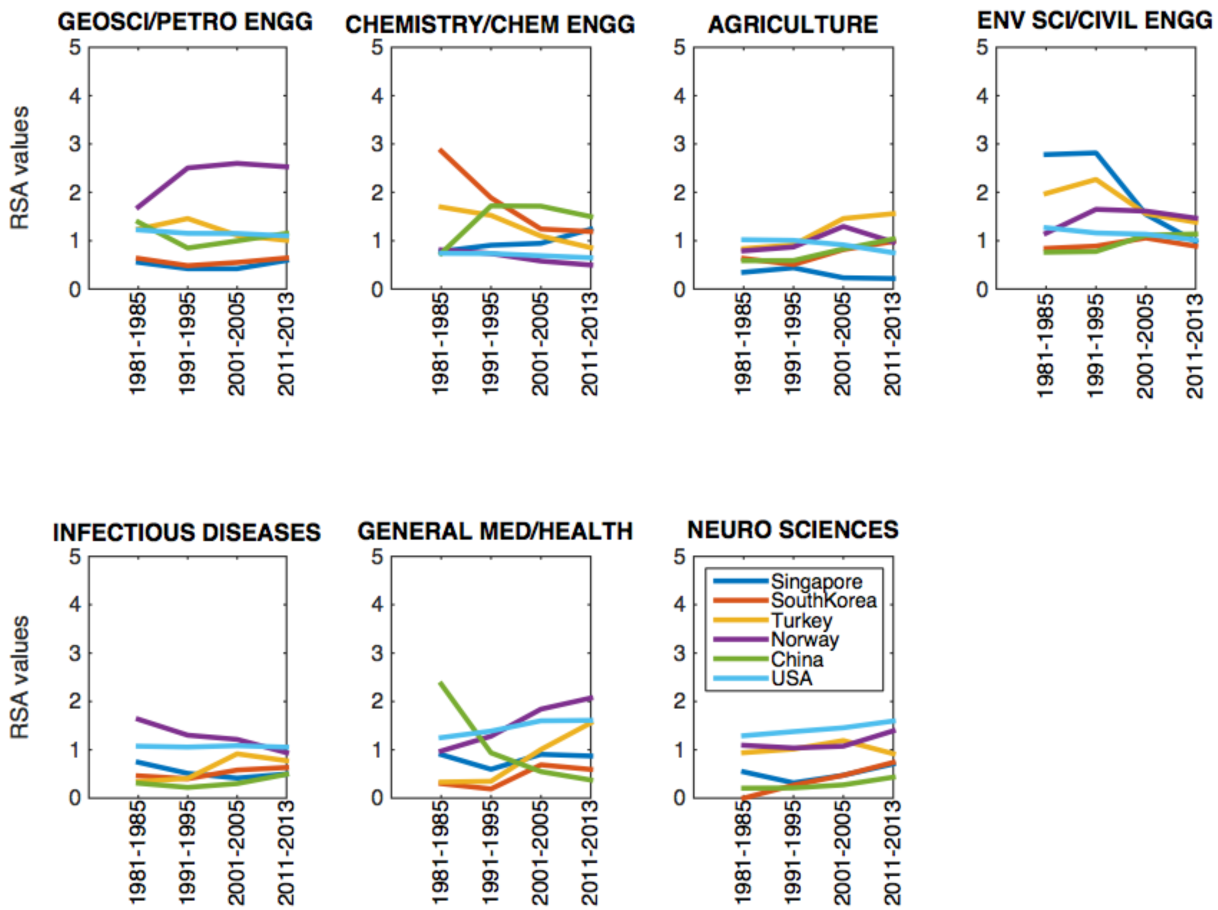

Fig. C. Norway shows high RSA in geological sciences/petroleum engineering and general medicine/health, and China in chemical engineering/chemistry. The US shows fairly even emphasis (RSA ~1) across most disciplines, with some higher levels for general medicine/health and neurosciences.

The changing patterns of specialization for the countries in the comparison group can be seen in Fig. B and C in this section. The trends for China show high initial focus (RSA value) in materials sciences, physics, and mathematics – confirming previous findings of sharp focus of specialization during early stages of development *(1)* – followed by falling levels of RSA indicating an evening of emphasis in national research across fields. An initial large focus in mechanical/industrial/aeronautical engineering, mathematics, and civil engineering/ environmental sciences is also evident for Singapore.

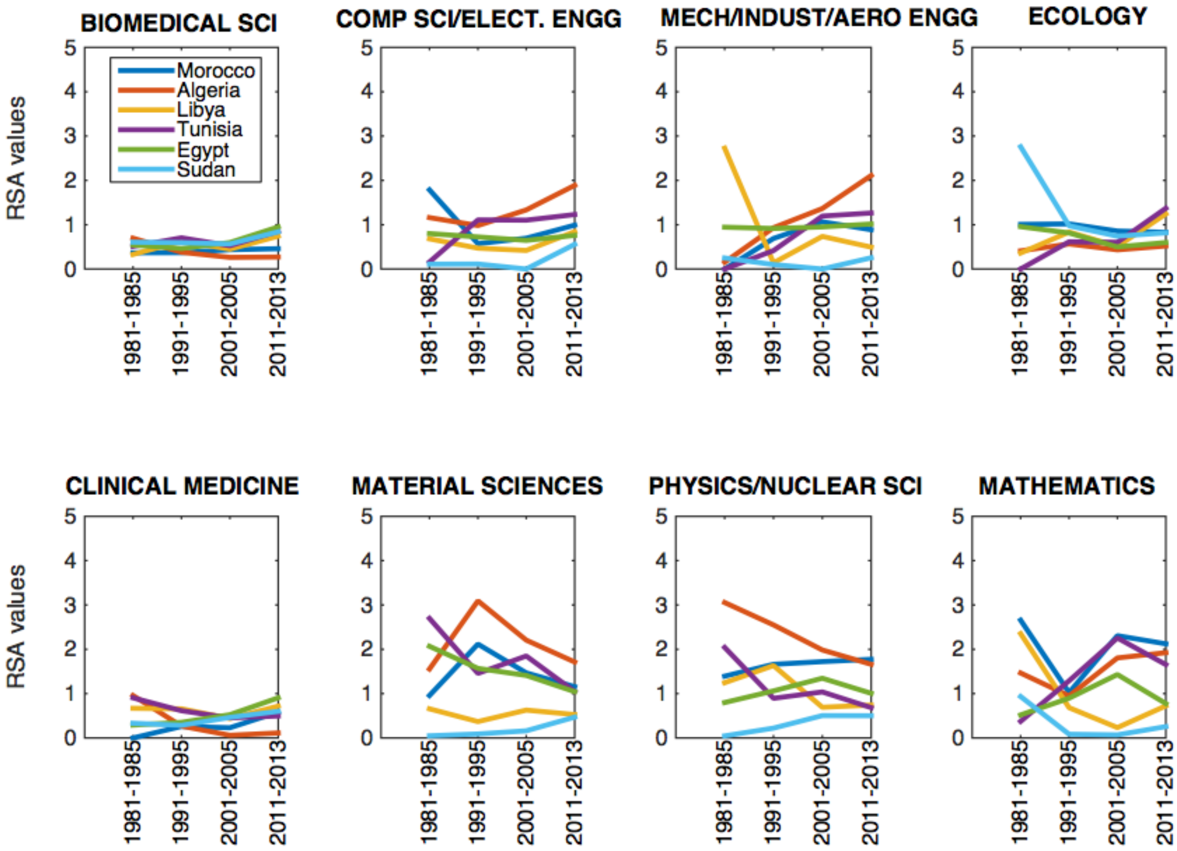

Figure D: RSA for countries in North Africa. There is low emphasis in biomedical sciences, clinical medicine, and ecology.

Algeria shows growing focus in computer science and electrical engineering, mechanical/industrial/aeronautical engineering, and somewhat sustained focus in materials science. Algeria, Tunisia, and Morocco show emphasis in mathematics and physics. Tunisia also shows growing focus in Agriculture (Fig. E). Egypt, the largest country in the group shows no particular specialization in any discipline.

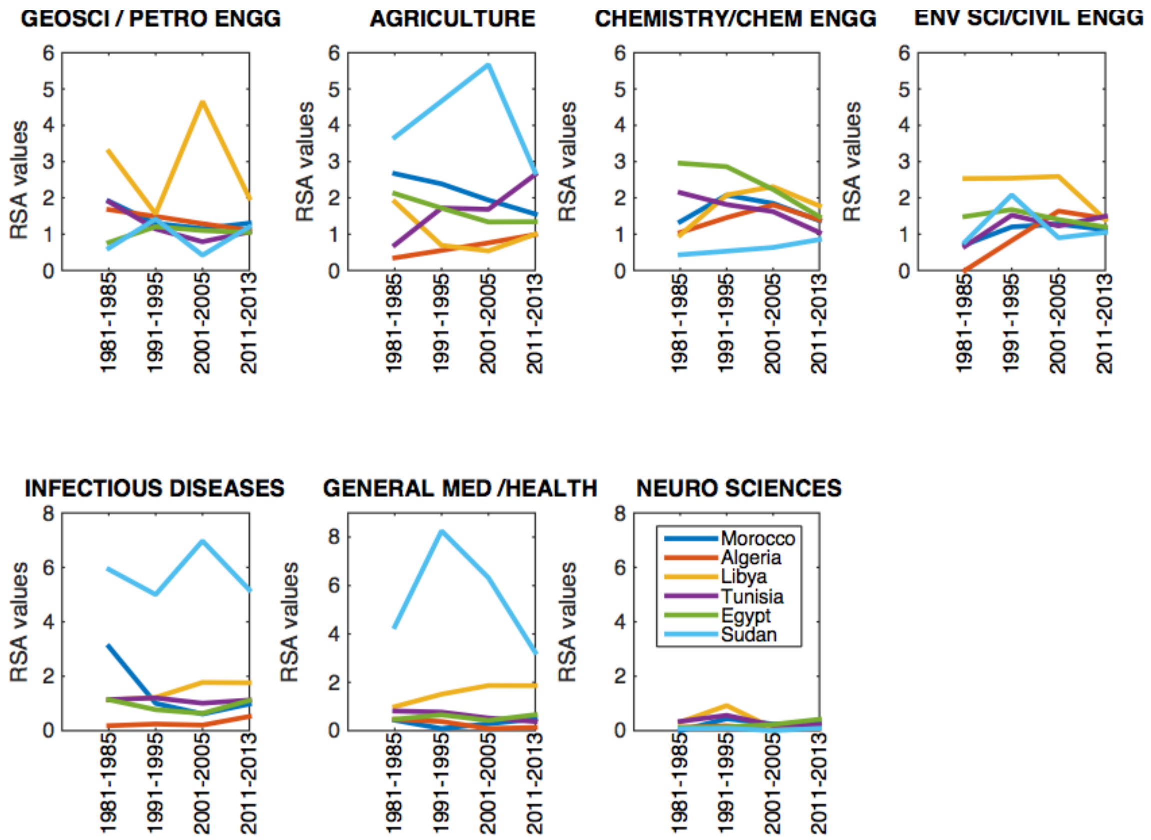

Figure E: RSA of North African countries.

Figures D and E collectively show that Egypt, the largest country in the group has no specific specialization, through agriculture, chemistry/chemical engineering, and civil engineering/environmental sciences show somewhat higher RSA values (~1.5). Sudan– a country with small scientific output – focused in agriculture, infectious diseases, and general medicine/health with RSA values up to 6 and 8 in some cases. Libya, another small country in terms of publications, showed strong emphasis (with RSA up to 5) in geological science/petroleum engineering (which also corresponds to the country's oil resources). It also shows higher RSA values (~2) in chemistry/chemical engineering, environmental science/civil engineering, infectious diseases, and general medicine/health.

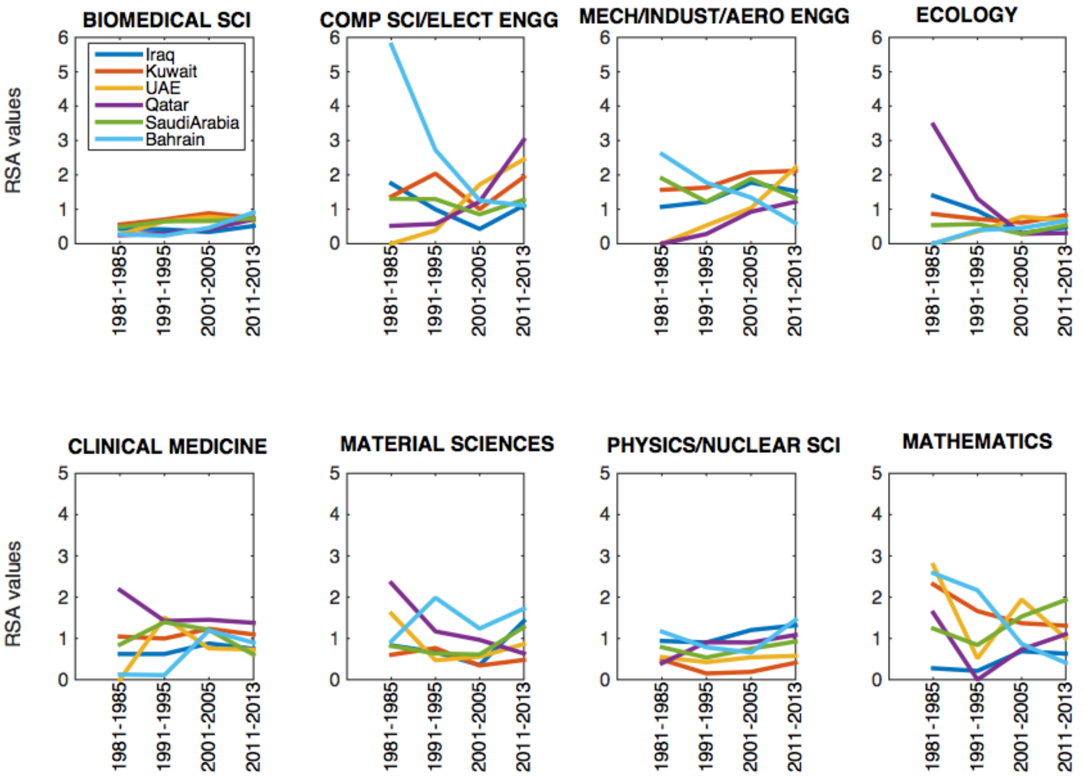

Fig. F: RSA values for Iraq and Gulf countries**.**

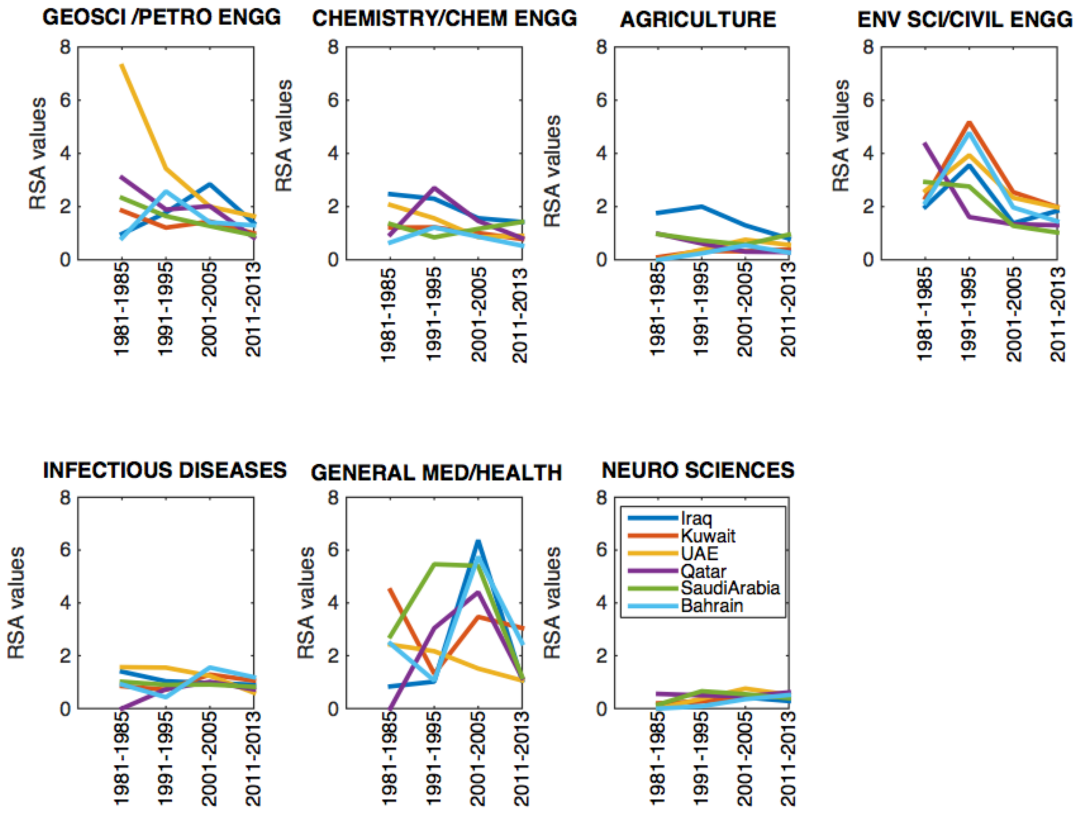

Fig. G: RSA values for Iraq and Gulf countries.

Bahrain, Qatar, and UAE the smaller countries, show some of the highest RSA (~4 – 7) in the 1981-1985 period. UAE and Qatar also show consistent rising trends in mechanical/industrial/aeronautical engineering, and in computer science/electrical engineering. Two specific patterns emerging concurrently across most of these countries are interesting: a rise in RSA for civil and environmental engineering in 1991-1995, and an increase in RSA in general medicine and health in 2001-2005 period. The trends in geological science/petroleum engineering and in chemical engineering/chemistry – subjects that relate closely to the largest economic and industrial sectors of oil and gas in these countries – are largely of falling RSA.

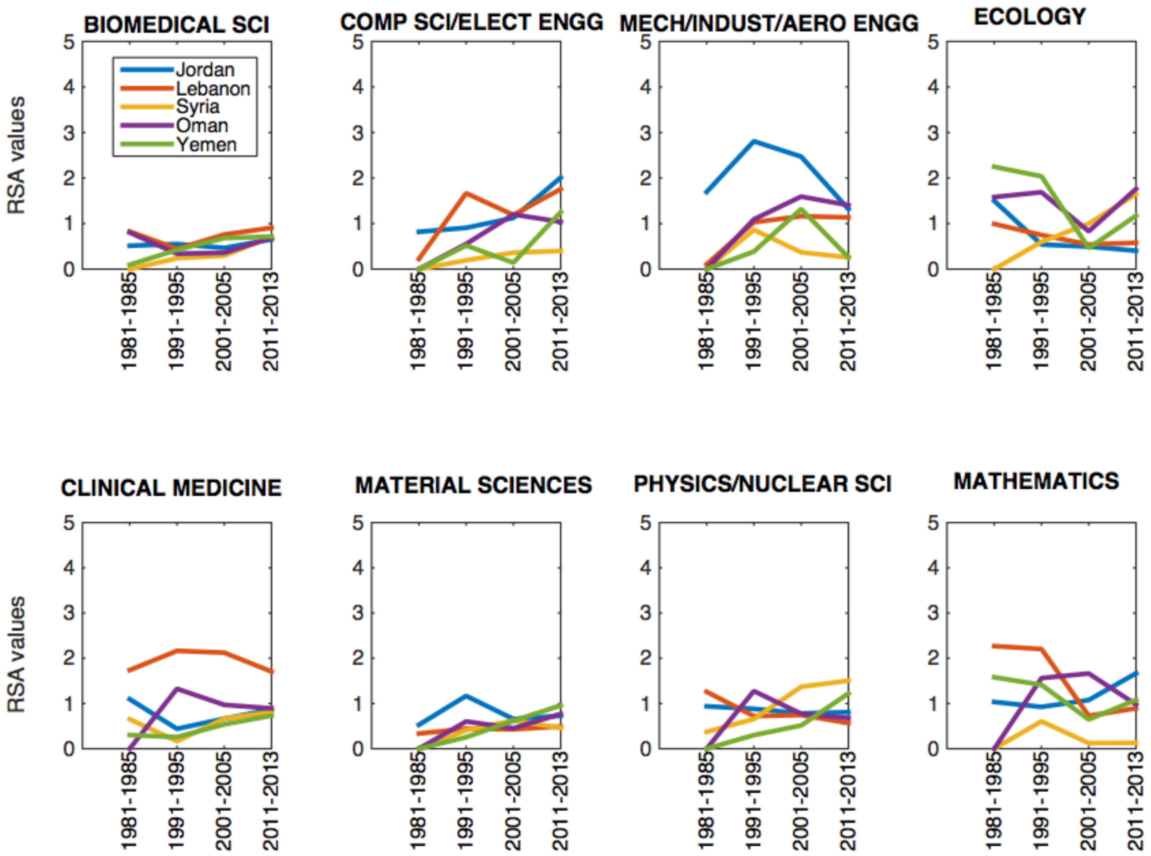

Fig. H: RSA for Jordan, Syria, Lebanon, Oman, and Yemen.

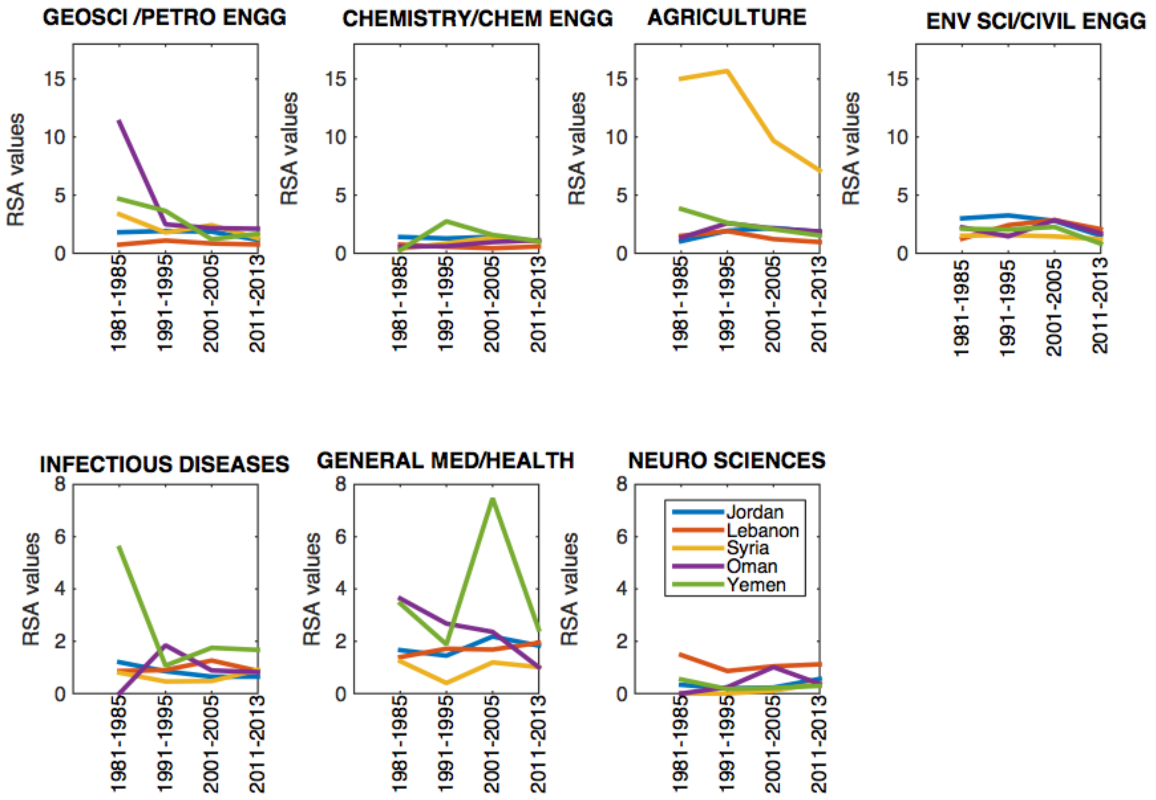

Fig. I: RSA for Jordan, Syria, Lebanon, Oman, and Yemen.

In this group of countries, the rising trends follow similar patterns to those observed for Gulf countries, with mostly growing emphasis in computer science/electrical engineering, and more modestly (but consistently) in biomedical sciences.

References and Notes:

1. R. M. May, The Scientific Wealth of Nations, *Science*, **275**, (5301), 793-796 (1997).
2. C. Freeman, *Technology Policy and Economic Performance: Lessons from Japan* (Pinter, London, 1987).
3. R. Solow, Technical Change and the Aggregate Production Function, *Review of Economics and Statistics*, **39**, 312–20 (1957)
4. J. A. Schumpeter, *The Theory of Economic Development* (Oxford University Press, 1934).
5. J. Adams, K. Christopher, P. Pendlebury, D. Hook, J. Wilson, "Exploring the changing landscape of Arabian, Persian, and Turkish research", (Global Research Report, Thompson Reuters, 2011).
6. A. Zewail, Dire need for a Middle Eastern science spring, *Nature Materials*, **13**, 38-320 (2014)
7. D. King, The Scientific Impact of Nations: What different countries get for their research spending, *Nature*, **430**, 311-316 (2004)
8. D. Hicks, P. Wouters, L. Waltman, S. de Rijcke, I. Rafols, Bibliometrics: The Leiden Manifesto for research metrics, *Nature*, **520**, 429-431 (2015).

9. R. C. Coile, Lotka's Frequency Distribution of Scientific Productivity, *Journal of the American Society for Information Science*, **28**(6), 366-370 (1977).
10. D. Hicks, J. Melkers in *Handbook on the Theory and Practice of Program Evaluation*. Eds. Al Link & Nick Vornatas. Edward Elgar (2012).
11. A. B. Jaffe, M. Trajtenberg, R. Henderson, Geographic localization of knowledge spillovers as evidenced by patent citations. *Quarterly Journal of Economics* **108**, 577–598 (1993).
12. C. Freeman, The 'National System of Innovation' in a historical perspective, Cambridge Journal of Economics, **19**, 5-24 (1995).
13. O. Sorenson, J. W. Rivkin, L. Fleming, Complexity, networks and knowledge flow. *Research Policy* **35**, 994–1017 (2006).
14. M. McKelvey, H. Alm, M. Riccaboni, Does co-location matter for formal knowledge collaboration in the Swedish biotechnology-pharmaceutical sector?, *Research Policy*, **32**, 483-501 (2003).
15. S. Wuchty, B. F. Jones, B. Uzzi, The Increasing Dominance of Teams in Production of Knowledge, *Science*, **316**, 1036-1039 (2007).
16. B. F. Jones, S. Wuchty, B. Uzzi, Multi-University Research Teams: Shifting Impact, Geography, and Stratification in Science, *Science*, **322**, 1259-1262 (2008).
17. J. Adams, The fourth age of research, *Nature*, **497**, 557-560 (2013).
18. J. P. Walsh, N. G. Maloney, Collaboration Structure, Communication Media, and Problems in Scientific Work Teams, Journal of Computer-Mediated Communication, **12**, 712-732 (2007).
19. B. Uzzi, J. Spiro, Collaboration and Creativity: The Small World Problem, *American Journal of Sociology*, **111** (2), 447-504 (2005)
20. "National Science Board Science and Engineering Indicators 2014", (NSB 14-01) National Science Foundation (2014).
21. L. Leydesdorff, I. Rafols, A Global Map of Science Based on the ISI Subject Categories, *Journal of the American Society for Information Science and Technology*, **60** (2) 348-362 (2009).
22. Hajjar D P, Moran G W, Siddiqi A, Richardson J E, Anadon L D, Narayanamurti V "Prospects for Policy Advances in Science and Technology in the Gulf Arab States: The Role for International Partnerships", *International Journal on Higher Education*, **3** (3), 45-57 (2014).
23. *Cochrane Handbook for Systematic Reviews of Interventions*, Version 5.1.0, Eds. J. Higgins, S. Green, (2011).
24. World DataBank, http://databank.worldbank.org/data/home.aspx.
25. http://images.webofknowledge.com/WOK46/help/WOS/h_advanced_fieldtags.html
26. C. A. Hidalgo, B. Klinger, A. L. Barabasi, R. Hausmann, The Product Space Conditions the Development of Nations, *Science*, 317, 482-487 (2007).
27. "Educating the Next Generation of Emiratis: A Master Plan for UAE Higher Education", United Arab Emirates Ministry of Higher Education and Scientific Research, Office of Higher Education Policy and Planning, (2007).
28. "Qatar National Development Strategy 2011~ 2016: Towards Qatar National Vision 2030", Qatar General Secretariat for Development Planning, Doha (2011).

29. M. Grueber, M., T. Studt, T., 2012 Global R&D Funding Forecast: R &D spending growth continues while globalization accelerates, *R & D Magazine*, December 16, (2011).

30. http://www.jadwa.com/en/download/2014-budget/saudi-arabias-2014-budget

31. "Kuwait University - Annual Report 2008-2009", Office of the Vice President for Research. Kuwait (2009).

32. Q. Schiermeier, Middle Eastern Promise, *Nature*, **500**, 111-112 (2013).

33. World Development Indicators: Population Dynamics, http://wdi.worldbank.org/table/2.1#

34. OCED Science, Technology and Industry Outlook (2012). http://www.oecd.org/sti/sti-outlook-2012-egypt.pdf

35. Y. Bhattacharjee, Saudi Universities Offer Cash in Exchange for Academic Prestige, *Science*, **334**, 1344-1345 (2011)
